# SOPHIE: Generative Neural Networks Separate Common and Specific Transcriptional Responses

**DOI:** 10.1016/j.gpb.2022.09.011

**Published:** 2022-10-07

**Authors:** Alexandra J. Lee, Dallas L. Mould, Jake Crawford, Dongbo Hu, Rani K. Powers, Georgia Doing, James C. Costello, Deborah A. Hogan, Casey S. Greene

**Affiliations:** 1 Genomics and Computational Biology Graduate Program, University of Pennsylvania, Philadelphia, PA 19104, USA; 2 Department of Microbiology and Immunology, Geisel School of Medicine at Dartmouth, Hanover, NH 03755, USA; 3 Department of Systems Pharmacology and Translational Therapeutics, University of Pennsylvania, Philadelphia, PA 19104, USA; 4 Wyss Institute for Biologically Inspired Engineering, Harvard University, Boston, MA 02115, USA; 5 Department of Pharmacology, University of Colorado School of Medicine, Denver, CO 80045, USA; 6 Center for Health AI, University of Colorado School of Medicine, Denver, CO 80045, USA; 7 Department of Biochemistry and Molecular Genetics, University of Colorado School of Medicine, Denver, CO 80045, USA

**Keywords:** Neural network, Transcriptomics, Software, Differential expression analysis, Machine learning

## Abstract

Genome-wide transcriptome profiling identifies genes that are prone to differential expression (DE) across contexts, as well as genes with changes specific to the experimental manipulation. Distinguishing genes that are specifically changed in a context of interest from common differentially expressed genes (DEGs) allows more efficient prediction of which genes are specific to a given biological process under scrutiny. Currently, common DEGs or pathways can only be identified through the laborious manual curation of experiments, an inordinately time-consuming endeavor. Here we pioneer an approach, Specific cOntext Pattern Highlighting In Expression data (SOPHIE), for distinguishing between common and specific transcriptional patterns using a generative neural network to create a background set of experiments from which a null distribution of gene and pathway changes can be generated. We apply SOPHIE to diverse datasets including those from human, human cancer, and bacterial pathogen *Pseudomonas aeruginosa*. SOPHIE identifies common DEGs in concordance with previously described, manually and systematically determined common DEGs. Further molecular validation indicates that SOPHIE detects highly specific but low-magnitude biologically relevant transcriptional changes. SOPHIE’s measure of specificity can complement log_2_ fold change values generated from traditional DE analyses. For example, by filtering the set of DEGs, one can identify genes that are specifically relevant to the experimental condition of interest. Consequently, these results can inform future research directions. All scripts used in these analyses are available at https://github.com/greenelab/generic-expression-patterns. Users can access https://github.com/greenelab/sophie to run SOPHIE on their own data.

## Introduction

Genome-wide transcriptomics analysis allows investigators to examine how global gene expression changes under the tested experimental stimulus or across different states, individuals, or genotypes. When interpreting the results of these analyses, attention tends to focus on controlling false discoveries [1], [2], [3], [4], *i.e*., differential gene expression patterns that arise due to noise or variation during measurement. In addition to false discoveries, however, certain genes tend to be commonly differentially expressed across a diverse panel of environmental stresses [5]. The response of this collection of genes was termed the environmental stress response (ESR). Despite the ESR being described more than two decades ago [5], compared to false discoveries, less attention has been paid to controlling for these common differentially expressed genes (DEGs). These findings include differential expression (DE) changes that are observed across experiments regardless of the experimental manipulation. Both gene-based [5], [6] and pathway-based [7] analyses can return common results.

While these common findings are not false discoveries, they provide little contextual information or insight into the biological process being queried as they are observed in many unrelated experiments. Not knowing which discoveries are common *vs.* specific can lead to misinterpretations or lack of specificity in interpreting results, so it is important to account for these different types of findings in addition to correcting for false discoveries.

Controlling for common findings is inordinately time-consuming and therefore limits the use of protocols that would identify them. Current methods rely on manual curation of a background set of experiments to select experiments with consistent experimental design and platform, as well as to use metadata to group samples for downstream statistical analysis. Re-curation is required to derive an appropriate background distribution in a new context, such as when switching to a new measurement platform, applying a different experimental design or analytical approach, incorporating new data, or examining a different organism. These background experiments are analyzed to identify genes and pathways that are common based on the frequency at which they are differentially expressed in the background experiments [6], [7]. Even when data are readily available, curating and analyzing hundreds of experiments requires a significant time investment to define a compendium of experiments to use as a background.

We introduce a general approach, termed Specific cOntext Pattern Highlighting In Expression data (SOPHIE), which distinguishes between common and specific transcriptional signals in a selected template experiment using a generative neural network [8] to simulate a set of background transcriptome experiments. In general, generative neural networks are a class of machine learning algorithms that model the distribution of the input data and thereby allow new data to be generated. Consequently, by using a generative neural network trained on existing transcriptomic data, we can generate a more realistic background distribution compared to assuming a normal distribution as a reference. These generative models also allow SOPHIE to automate the analysis of common DEGs since this approach requires enough gene expression data to generate synthetic measurements; however, the data do not need to be curated by experimental design, which removes a usually time-consuming step. Such data are readily available through National Center for Biotechnology Information (NCBI) Gene Expression Omnibus (GEO) [9], Short Read Archive (SRA) [10], European Nucleotide Archive (ENA) [11], and other repositories. Many datasets are already processed for reuse through projects such as recount2 [12] or ARCHS4 [13]. Because SOPHIE relies on generating synthetic data that match a user-selected template experiment, it can be applied to arbitrary downstream analytical workflows, which could be DE analysis, pathway analysis, or other methods, to provide a background distribution of common findings. Furthermore, by using a single template experiment, we only need to define sample groupings for this one experiment as opposed to manually annotating groups for hundreds of experiments. Overall, without the need for manual curation to define a compendium and group samples, SOPHIE can expand lists of genes for follow-up by identifying genes that are context-specific but have subtle signals and are thus understudied in that context. SOPHIE can also filter lists of genes for functional validation by limiting a list of genes to those that are both differentially expressed and highly specific. Overall, SOPHIE’s specificity score can be a complementary indicator of activity compared to the traditional log_2_ fold change (FC) measure and can help drive future analyses.

We use SOPHIE to identify common DEGs in a human microarray dataset, and the results are consistent with the prior manually curated report using the same human microarray dataset. Next, we find consistent common DEGs using a different human microarray dataset, a cancer cell line dataset, demonstrating that common DEGs are shared across contexts. Furthermore, we also find consistent common DEGs using human RNA-seq data, demonstrating that common DEGs are shared across platforms too. SOPHIE is also generalizable across organisms as shown by its application to the opportunistic bacterial pathogen and model organism, *Pseudomonas aeruginosa*. The metabolic choices of *P. aeruginosa* can impact its pathogenicity, and using SOPHIE to analyze alternative carbon utilization in *P. aeruginosa* [14] reveals gene expression changes that are specific to different regulatory levels in the hierarchy of the carbon catabolite repression cascade. This analysis reveals context-specific regulation of arginine metabolism, whose genes would be undetected in a traditional DE analysis due to their low magnitude. Based on our SOPHIE results, we hypothesize that these arginine-related gene expression changes are specific to some but not all gene perturbations in the carbon catabolite repression pathway that controls alternative carbon utilization. Experimental data support the prediction that arginine catabolism is specifically perturbed by some, but not all mutations of genes, in the pathway. This demonstrates that SOPHIE can successfully identify candidate genes that are specifically relevant to the context of interest but difficult to uncover through previously developed analysis tools.

## Method

### Gene expression datasets

We used four complementary gene expression compendia in this work. Three were sets of assays of human samples, two via microarray and the other via RNA-seq profiling. The fourth was a collection from the microbe *P*. *aeruginosa*.

The first human compendium that we used contained gene expression data from the study by Crow et al. [6], named as Crow et al. compendium. We downloaded the dataset from Gemma (March 20, 2021). Gemma contains public gene expression data primarily from GEO. These samples were measured on the GPL570 (Affymetrix human genome U133 plus 2.0 array) platform, testing at least one condition and reporting at least one DEG. Samples were processed using the rma library to convert probe intensity values from the .cel files to log_2_ gene expression measurements, and these gene expression values were then log_10_ transformed to account for the large spread of the data and then normalized to a range of 0–1 per gene. We also removed a subset of genes and samples that contained NaNs, where the data were not available. This resulted in an expression matrix that contained 7130 genes and 32,082 samples.

The second human compendium that we used contained gene expression data from the study by Powers et al. [7], named Powers et al. compendium. We downloaded the dataset from synapse on October 7, 2020. This dataset contained samples from the GEO measured on Affymetrix human genome U133 plus 2.0 array. Samples were selected based on the following criteria: having at least two replicates per condition and containing a vehicle control. The dataset included 442 experiments testing the response of small-molecule treatments in cancer cell lines. Samples were processed using the rma library to convert probe intensity values from the .cel files to log_2_ gene expression measurements, and these gene expression values were then normalized to a range of 0–1 per gene. This resulted in an expression matrix that contained 6763 genes and 2410 samples.

The third human compendium that we used included human RNA-seq data from recount2 [12], named as recount2 compendium. We downloaded all SRA data in recount2 as RangedSummarizedExperiment (RSE) objects for each project ID using the recount library in Bioconductor (v1.12.0). Raw reads were mapped to genes using Rail-RNA [15], which included exon-exon splice junctions. Each RSE contained counts summarized at the gene level using the GENCODE v25 (GRCh38.p7, CHR) annotation provided by GENCODE [16]. These RSE objects include coverage counts as opposed to read counts, so we applied the scale_counts function to scale by sample coverage (average number of reads mapped per nucleotide). The compendium contained 49,651 samples with measurements for 58,129 genes. Our goal was to compare percentiles with ones provided by Crow et al. [6], which required us to map the Ensemble gene IDs in recount2 to HUGO Gene Nomenclature Committee (HGNC) symbols. We used the intersection of genes between the recount2 set and set from Crow and colleagues [6]. This resulted in a gene expression matrix of 49,651 samples and 17,755 genes. We then normalized gene expression values to a range of 0–1 per gene. This recount2 compendium contained a heterogeneous set of gene expression experiments — 31 tissue types (*e.g.*, blood and lung), 57 cell types (*e.g.*, stem and HeLa), and multiple experimental designs (*e.g.*, case-control and time-series).

The last compendium contained *P. aeruginosa* gene expression data that were collected and processed as described by Lee et al. [8], named as *P. aeruginosa* compendium. The dataset was originally downloaded from the Analysis using Denoising Autoencoders of *g*ene Expression (ADAGE) [17] GitHub repository (https://github.com/greenelab/adage/tree/master/Data_collection_processing). Raw microarray data (measured on the release of the GeneChip *P. aeruginosa* genome array and the time of data freeze in 2014) were downloaded as .cel files. Then rma was used to convert probe intensity values from the .cel files to log_2_ gene expression measurements. These gene expression values were then normalized to a range of 0–1 per gene. The resulting matrix contained 989 samples and 5549 genes that represent a wide range of gene expression patterns including characterization of clinical isolates from cystic fibrosis infections, differences between mutant and wild type (WT), response to antibiotic treatment, microbial interactions, and the adaptation from water to gastrointestinal (GI) tract infection.

Given that these compendia contain a heterogeneous collection of experiments, batch effects are certainly present. However, previously examinations of the effects of technical sources of variability in a large-scale compendium setting suggest that when many experiments are combined, batch correction isn’t necessary and can even be harmful [8]. Our compendia fall into this category, so we have not batch corrected.

### SOPHIE

#### Simulate gene expression experiments using ponyo

Our simulation applied the experiment-level simulation approach from Lee and colleagues [8]. The configuration of the variational autoencoder (VAE) we used was the same as in this previous publication — 2500 features in the hidden layer and 30 latent space features. Each layer used a rectified linear unit (ReLU) activation function to combine weights from the previous layer. We performed a 75:25 split of the data for training and validation. The hyperparameters were manually adjusted based on a visual inspection of the validation loss outputs. Our optimal hyperparameter settings were: a learning rate of 0.001, a batch size of 10, and warmups set to 0.01. We trained three VAE models using the Crow et al. (10 epochs), recount2 (40 epochs), Powers et al. (40 epochs), and *P. aeruginosa* (100 epochs) compendia.

We selected a template experiment from each compendium (SRP012656 from recount2, GSE10281 from Crow et al., GSE11352 from Powers et al., and E-GEOD-33245 from *P. aeruginosa*). For the most part, the selected template experiments are assumed to come from a similar distribution as our background compendium. We simulated a new experiment by linearly shifting the selected template experiment to a new location in the latent space. This new location was randomly sampled from the distribution of the low dimensional representation of the trained gene expression compendium. The vector that connects the centroid of the template experiment and the new location was added to all samples of the template experiment to create a new simulated experiment. This process was repeated 25 times to create 25 simulated experiments based on the single template experiment. In general, we found that downstream statistical results were robust to different numbers of simulated experiments, so we used 25 experiments to compromise on the runtime of the downstream analyses (Figure S1).

#### DE analysis

For the recount2 compendium, we used the DESeq module in the DESeq2 library [18] to calculate DE values for each gene by comparing the two different conditions in the selected template experiment (SRP012656). The template experiment contained primary non-small cell lung adenocarcinoma tumors and adjacent normal tissues of 6 never-smoker Korean female patients. The DE analysis compared tumor *vs.* normal. Following a similar procedure for the array-based datasets (Crow et al. compendium, Powers et al. compendium, and *P. aeruginosa* compendium), we used the eBayes module in the *limma* library [19] to calculate differential gene expression values for each gene. The output statistics included log_2_ FC between the two conditions tested and *P* values adjusted by Benjamini–Hochberg’s method to control for false discovery rate (FDR). The template experiment we used for the Crow et al. compendium was GSE10281, which examined the expression profiles of breast cancer cells treated with letrozole. The template experiment we used for the Powers et al. compendium was GSE11352, which examined the transcriptional response of MCF7 breast cancer cells to estradiol treatment. Thus, the DE analysis compared samples untreated *vs.* treated. The template experiment we used for the *P. aeruginosa* compendium was E-GEOD-33245, which contained multiple comparisons examining the CbrAB system. The two we focused on for our analysis compared WT *vs.* Δ*cbrB* and Δ*crc* mutants in lysogeny broth (LB) media.

For the *P. aeruginosa* experiment, DEGs were those with FDR-adjusted cutoff < 0.05 (using Benjamini–Hochberg correction) and |log_2_FC| > 1, which are thresholds frequently used in practice.

#### Calculate the specificity of each gene (z-score)

Using the association statistics from the DE analysis, we calculated a score to indicate if a gene was specifically differentially expressed in the template experiment. We calculated a z-score for each gene as follows: z-score of gene *A* = [log_2_ FC gene *A* in template experiment − mean (log_2_ FC gene *A* in simulated experiments)]/*var* (log_2_ FC gene *A* in simulated experiments).

A higher z-score indicated that a gene was specifically differentially expressed in the template experiment in reference to the null set of experiments (*i.e.*, 25 simulated experiments). This z-score was meant to guide scientists to select genes of interest. Genes could be selected solely based on z-scores (*i.e.*, selecting genes with the highest z-score), or there could be additional constraints that are used to select genes in combination with z-scores.

### SOPHIE *vs.* traditional DE analysis

The goal of this analysis was to determine how often SOPHIE distinguishes between specific and common DEGs (*i.e.*, ranking specific genes higher than common genes) compared to using traditional DE analysis. The approach was to simulate data where we manually determined which genes were specific and which ones were common genes.

First, we created a training compendium for SOPHIE. This compendium was composed of 90 perturbation experiments. Each experiment contained 8 samples, 4 perturbed and 4 control, with 1000 genes. The expression profile for each gene was determined by sampling from a negative binomial distribution, where the success rate varied between different genes. We randomly selected 100 of the 1000 genes to be common genes. We also selected 10 of the remaining 900 non-common genes to be specific genes. For each experiment, the same scaler value was added to the 100 common and 10 specific genes of the 4 perturbed samples. This scaler value was determined based on the expression of the simulated experiment — the scaler value was the 98th quantile of the median expression of genes in the simulated experiment. This process was repeated 90 times to generate 90 total experiments with 720 samples and 1000 genes, where each experiment had a different set of 10 specific genes that were scaled. Next, we generated a template experiment following the same procedure. Then we applied SOPHIE using the template experiment, which yielded a z-score for each gene. We ranked genes based on their z-scores, such that higher ranks corresponded to higher z-scores which indicated that the gene is more specific. Likewise, we applied traditional DE analysis for each template which yielded a log_2_ FC value for each gene. Again, we ranked genes based on their log_2_ FC values, such that higher ranks corresponded to higher log_2_ FC values, which indicated that the gene is more specific. Finally, plotted the ranking of the common and specific genes obtained using traditional DE analysis and SOPHIE.

### Enrichment analysis

The goal of enrichment analysis (EA) was to detect coordinated changes in prespecified sets of related genes, *i.e.*, those genes in the same pathway or sharing the same Gene Ontology (GO) term.

Our primary method was Gene Set Enrichment Analysis (GSEA), for which we used the fgsea module from the fgsea library [20], [21]. The method first ranked all genes based on the DE association statistics. In this case, we used the log_2_ FC. An enrichment score (ES) is defined as the maximum distance from the middle of the ranked list. Thus, the ES indicates whether the genes contained in a gene set are clustered toward the beginning or the end of the ranked list (indicating a correlation with the change in expression). The statistical significance of the ES is estimated by a phenotypic-based permutation test to produce a null distribution for the ES (*i.e.*, scores based on permuted phenotype). Each pathway was output with statistics including a Benjamini–Hochberg adjusted *P* value. The pathways used in this analysis were the hallmark pathways for the Powers et al. compendium.

Other methods we used included: gene set variation analysis (GSVA) [22], correlation adjusted mean rank gene set test (CAMERA) [23], and over-representation analysis (ORA). GSVA is a self-contained gene set test that estimates the variation of gene set enrichment over the samples independent of any class label. We used the gsva function from the gsva library. CAMERA is a competitive gene set test that performs the same rank-based test procedure as GSEA but also estimates the correlation between genes instead of treating genes independently. For CAMERA, we used the camera function that is part of the *limma* library [24]. Last, ORA is a method that uses the hypergeometric test to determine if there is a significant over-representation of a pathway in the selected set of DEGs. Here we used the clusterProfiler [25] library but there are multiple options for this analysis.

### Comparison of gene percentiles

We wanted to compare the percentile of human genes identified using SOPHIE (trained on Crow et al., Powers et al., and recount2 datasets) with the percentile found from the study by Crow et al. [6], which identified a set of genes as common DEGs based on how frequently they were found to be differentially expressed across 635 manually curated experiments. In their study, they ranked genes as 0 if they were not commonly differentially expressed and 1 if they were commonly differentially expressed. Our genes were ranked from 1 to 17,754 based on their median |log_2_ FC| value across the 25 simulated experiments. We linearly scaled the gene ranks to be a percentile from 0 to 100. Finally, we applied Spearman correlation to compare the percentile for each gene.

We performed this same correlation analysis comparing SOPHIE trained on the *P. aeruginosa* compendium with percentiles generated from the GAUGE-annotated *Pseudomonas aeruginosa* and *Escherichia coli* transcriptomic compendia for reanalysis (GAPE) project from the Stanton lab (https://github.com/DartmouthStantonLab/GAPE) [26]. The GAPE dataset contained ANOVA statistics generated for 73 *P. aeruginosa* microarray experiments using the Affymetrix platform GPL84. We downloaded the DE statistics for 73 array experiments from the associated repository (https://github.com/DartmouthStantonLab/GAPE/blob/main/Pa_GPL84_refine_ANOVA_List_unzip.rds). For each experiment, we identified DEGs using |log_2_ FC| > 1 and FDR-adjusted *P* < 0.05. We then calculated the percentile per gene based on the proportion that they were found to be differentially expressed. We compared these GAPE percentiles against those found by SOPHIE.

We also compared percentiles of genes amongst two SOPHIE-generated results. This included comparison of percentiles generated from two SOPHIE runs using the same template experiment and comparison of percentiles generated by SOPHIE using two different template experiments.

### Comparison of pathway percentiles

We wanted to compare the percentile of pathways identified using SOPHIE (trained on Powers et al., Crow et al., and recount2 datasets) with the percentile based on the data from the study by Powers and colleagues [7]. There was no pathway ranking provided in their publication, so we defined a reference ranking by calculating the fraction of the 442 experiments that a given pathway was found to be significant (FDR-adjusted *P* < 0.05 using Benjamini–Hochberg method), and used these to rank pathways and then converted the ranking to a percentile as described above. We used the hallmarks_qvalues_GSEAPreranked.csv file from https://www.synapse.org/#!Synapse:syn11806255. The file contains the *Q* values for the test: given the ES of the experiment is significant compared to the null distribution of ESs, where the null set is generated from permuted gene sets. Our percentile is based on the median Benjamini–Hochberg adjusted *P* value across the simulated experiments. We compared our percentile with the reference percentile using the Spearman correlation. We only showed the comparison of SOPHIE trained on Powers et al., but not Crow et al. or recount2.

### Latent variable analysis

The goal of this analysis was to examine why genes were found to be commonly differentially expressed — we sought to answer the question: are common DEGs found in more pathway-level information extractoR (PLIER) latent variables (LVs) [27] compared to specific genes? The PLIER model performed a matrix factorization of the same recount2 gene expression data to get two matrices: loadings (Z) and latent matrix (B). The loadings (Z) were constrained to aligned with curated pathways and gene sets specified by prior knowledge to ensure that some but not all LVs capture known biology. For this analysis, we focused on the Z matrix, which is a weight matrix that has dimensions of 6750 genes by 987 LVs. For this analysis, common DEGs were at and above the 60th percentile (approximately the top 40% of genes were selected based on the distribution of weights) using SOPHIE trained on recount2. We calculated the coverage of common DEGs *vs.* other genes across these PLIER LVs. For each gene we calculated two values: 1) how many LVs in which the gene was present (*i.e.*, having a non-zero weight score according to the Z matrix); 2) how many LVs in which the gene had a high weight score, using the 98th percentile for the LV distribution as the threshold.

### Network analysis

In order to examine associations between common DEGs and pathways or functional modules in *P. aeruginosa*, we constructed a network of gene–gene interactions. Nodes in this network represent *P. aeruginosa* genes, and edges represent correlations between the ensemble ADAGE (eADAGE) weight vectors of the two genes they connect. We constructed the network using the ADAGEpath R package, described in more detail in the associated manuscript [17]. To form the final network, we removed all edges (correlations) with a value between −0.5 and 0.5, and took the absolute value of the remaining edges (so negative edge weights became positive).

There are many existing methods to partition a network into well-connected, non-overlapping subnetworks, often referred to as communities. Using our gene similarity network, we sought to answer the question: do common DEGs tend to occupy fewer network communities than a similar set of random genes, or do they tend to spread out across comparatively many communities? We chose two representative methods to divide the network into communities: 1) the Louvain method [28], as implemented in the python-igraph package [29], and 2) the “planted partition” model [30] (data not shown), as implemented in the graph-tool Python package [30]. In order to make a meaningful comparison between common and non-common DEGs, we sampled an equal number of both gene categories. This meant that the non-common DEGs were approximately degree-matched with the common DEGs (*i.e.*, for each commonly changed gene we sampled a specific DEG with approximately the same network degree). We performed this sampling procedure 1000 times. We then counted the number of communities containing at least one commonly changed gene and compared this count to the distribution across the 1000 samples of the number of communities containing at least one sampled non-commonly changed gene.

In addition, we used the same eADAGE gene similarity network to compute several metrics describing individual network nodes, which we then compared between common and non-common DEGs. For both sets of genes, we calculated: 1) node degree, 2) edge weight, 3) betweenness centrality [31], and 4) PageRank centrality [32]. For each of these metrics, we used the implementations in the graph-tool Python package. In contrast to the other metrics, betweenness centrality treats edge weights as “costs” (lower = better, as opposed to correlation or similarity measures where higher = better), so for the betweenness centrality calculation, we transformed all edge weights by setting edge cost = 1 − correlation.

### Strain construction

Plasmids for making in-frame deletions of *cbrB* and *crc* were made using a *Saccharomyces cerevisiae* recombination technique previously described [33]. The arabinose-inducible *cbrB* expression vector was made using Gibson cloning [34]. All plasmids were sequenced at the Molecular Biology Core at the Geisel School of Medicine at Dartmouth and maintained in *E. coli*. In frame-deletion constructs were introduced into *P. aeruginosa* by conjugation via S17/lambda pir *E. coli*. Merodiploids were selected by drug resistance and double recombinants were obtained using sucrose counter-selection and genotype screening by Polymerase Chain Reaction (PCR). The *cbrB* and empty expression vectors were introduced into *P. aeruginosa* by electroporation and selected by drug resistance.

### *P. aeruginosa* experiment

Bacteria were maintained on LB with 1.5% agar. For strains harboring expression plasmids, 300 μg/ml carbenicillin or 60 μg/ml gentamycin was added. Yeast strains for cloning were maintained on yeast peptone dextrose (YPD) with 2% agar. Planktonic cultures (5 ml) were grown on roller drums at 37 °C from single colonies for 16 h in LB (under antibiotic selection for the appropriate strains). The 16 h LB cultures were normalized to OD_600 nm_ = 1 in 2 ml, and a 250 µl aliquot of the normalized culture was used to inoculate three 5 ml cultures of M63 medium containing 10 mM arginine as a sole carbon source under inducing conditions (0.2% arabinose) for a starting OD_600 nm_ = 0.05. Inoculated cultures were grown at 37 °C on the roller drum and cellular density (OD_600 nm_) was monitored using a Spec20 every hour for 8 h. Each data point is representative of the average of the 3 replicates per day for 3 independent days.

## Results

### SOPHIE distinguished between common and specific transcriptional patterns

**Figure 1:**
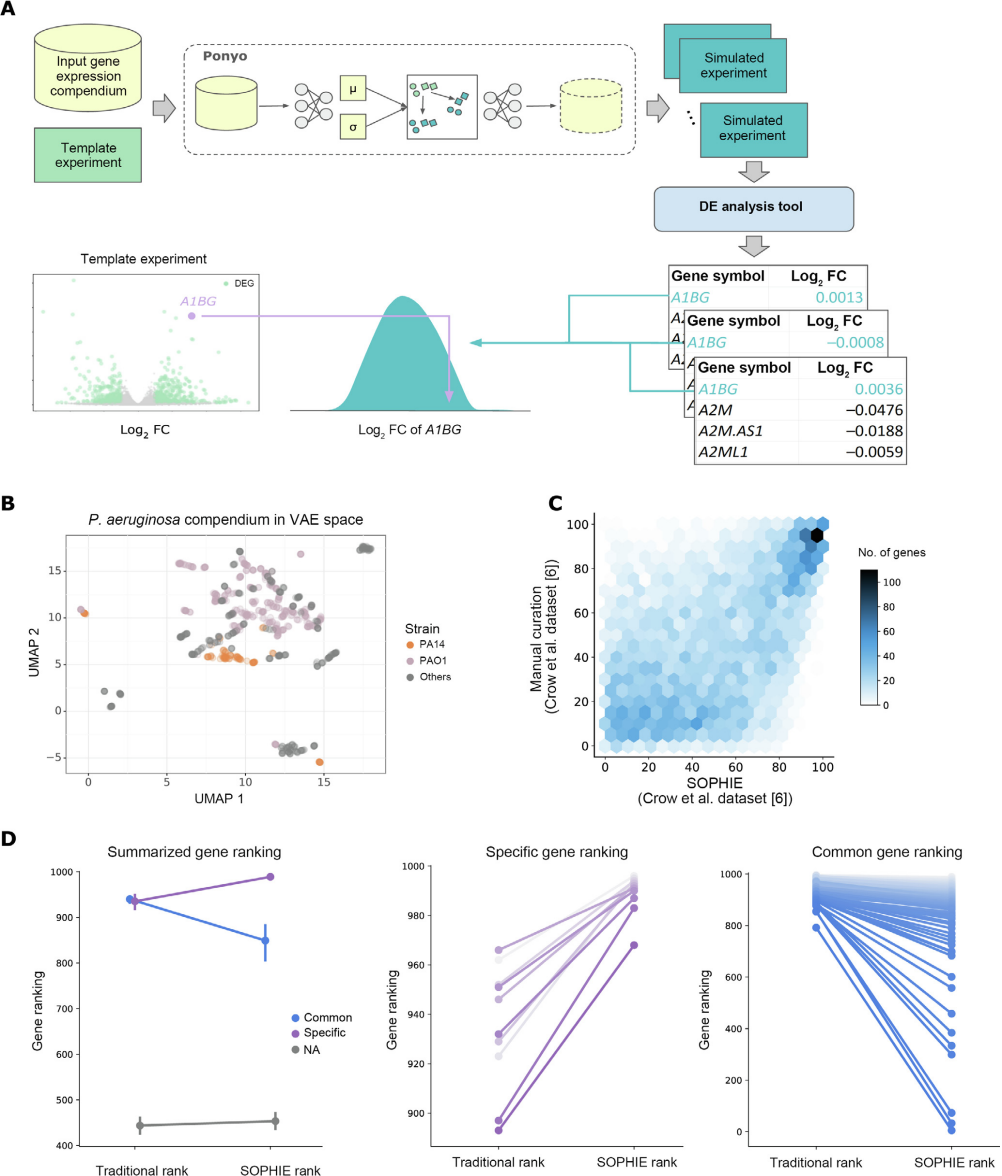
**Overview of the SOPHIE workflow** SOPHIE is an approach to distinguishing between common and specific DEGs using a generative neural network. **A.** SOPHIE workflow is designed to distinguish between common and specific transcriptional signals. SOPHIE starts by applying ponyo to simulate gene expression experiments. Next, SOPHIE applies DE analysis tools like DESeq2 for RNA-seq data or *limma* for array data to get association statistics for each simulated experiment. Finally, SOPHIE returns a distribution of how changed each gene is across the collection of background simulated experiments so that users can compare gene expression changes from their template experiment of interest. **B.** UMAP projection of the *P. aeruginosa* compendium encoded in the VAE latent space revealed clusters of strain types (PAO1 and PA14). **C.** Spearman correlation (R^2^ = 0.55) between gene percentiles obtained by our SOPHIE approach trained on the Crow et al. dataset (array) [6] using GSE10281 as a template (x-axis) and gene percentiles obtained by manually curated experiments from the same Crow et al. dataset [6] (y-axis). **D.** Difference between the mean ranking of specific genes and common genes using SOPHIE and traditional DE analysis. Using a Wilcoxon signed-rank test, the distribution of the ranking of common genes was significantly less (*P* = 1E−17) in SOPHIE compared to the traditional approach. Similarly, the distribution of ranking of specific genes was significantly greater (*P* = 0.0025) in SOPHIE compared to the traditional approach. NA indicates all other genes, *i.e.*, neither common or specific. SOPHIE, Specific cOntext Pattern Highlighting In Expression data; DEG, differentially expressed gene; DE, differential expression; FC, fold change; UMAP, Uniform Manifold Approximation and Project; VAE, variational autoencoder.

The main steps for SOPHIE are illustrated in Figure 1A. The first step is to generate a background set of transcriptome experiments, for which we applied ponyo [8]. Ponyo uses a generative neural network, in this case, a VAE, to generate new samples that match a selected template experiment’s design (in our case the experiment is comprised of a control and one experimental group). This VAE model appeared to separate samples by biological features, such as by *P. aeruginosa* strain types (Figure 1B). Using this VAE model, new samples are generated by encoding the template samples and linearly shifting them in the latent space while preserving their relative positioning. Intuitively, this latent space translation is akin to simulating an experiment with the same experimental design but studying a different biological process or a different set of conditions. SOPHIE uses ponyo to simulate realistic-looking transcriptome experiments that serve as a background set for distinguishing between common and specific transcriptional signals.

For the next step, SOPHIE applies a DE analysis tool, like DESeq or *limma*, to get association statistics. Then those DE statistics are used to rank genes by their propensity to be differentially expressed, which we then use to interpret the changes observed in a template experiment. This allows investigators to distinguish common DEGs from context-specific ones in their results. We generate a z-score per gene to capture the relationship between a gene’s magnitude of change in the template experiment compared to the background distribution. In general, if a gene’s magnitude of change is larger than the mean change in the background distribution, this gene is considered specific. However, the specificity threshold will depend on the experiment of interest and what additional contextual constraints are being considered.

### Common DEGs identified by the simulation-based approach recapitulated the curation-derived ones

Identifying common DEGs has been challenging because it requires extensive manual curation. We sought to compare the common DEGs identified by SOPHIE with those identified in the study by Crow and colleagues [6]. This previous study curated 2456 human microarray datasets from the GPL570 (Affymetrix human genome U133 plus 2.0 array) platform to identify common DEGs [6]. This study provided a list of genes ranked based on how frequently they were identified as differentially expressed across approximately 600 experiments (here referred to as the Crow et al. results). We compared the SOPHIE-predicted common DEGs using a VAE trained on the Crow et al. dataset with the Crow et al. results. We calculated the percentile of genes by their median log_2_ FC across the 25 simulated experiments. Comparing the gene percentiles from the Crow et al. results to our SOPHIE results revealed substantial concordance (Figure 1C; Spearman correlation coefficient = 0.55). There was also a significant (*P* < 1E−16) over-representation of SOPHIE-identified common DEGs within the common DEGs identified by Crow and colleagues [6]. SOPHIE recapitulated the primary results of the Crow et al. results identified by the curation-based approach. While the Crow et al. results relied on having a manually curated dataset, SOPHIE identified these genes in a more scalable and automated way, leveraging existing gene expression data to simulate a background set of experiments to use as a reference. Additionally, compared to using a traditional DE analysis, SOPHIE better distinguished between specific and common genes based on the ranking of specific and common DEGs (Figure 1D).

### SOPHIE identified common DEGs that were consistent across contexts and platforms

**Figure 2:**
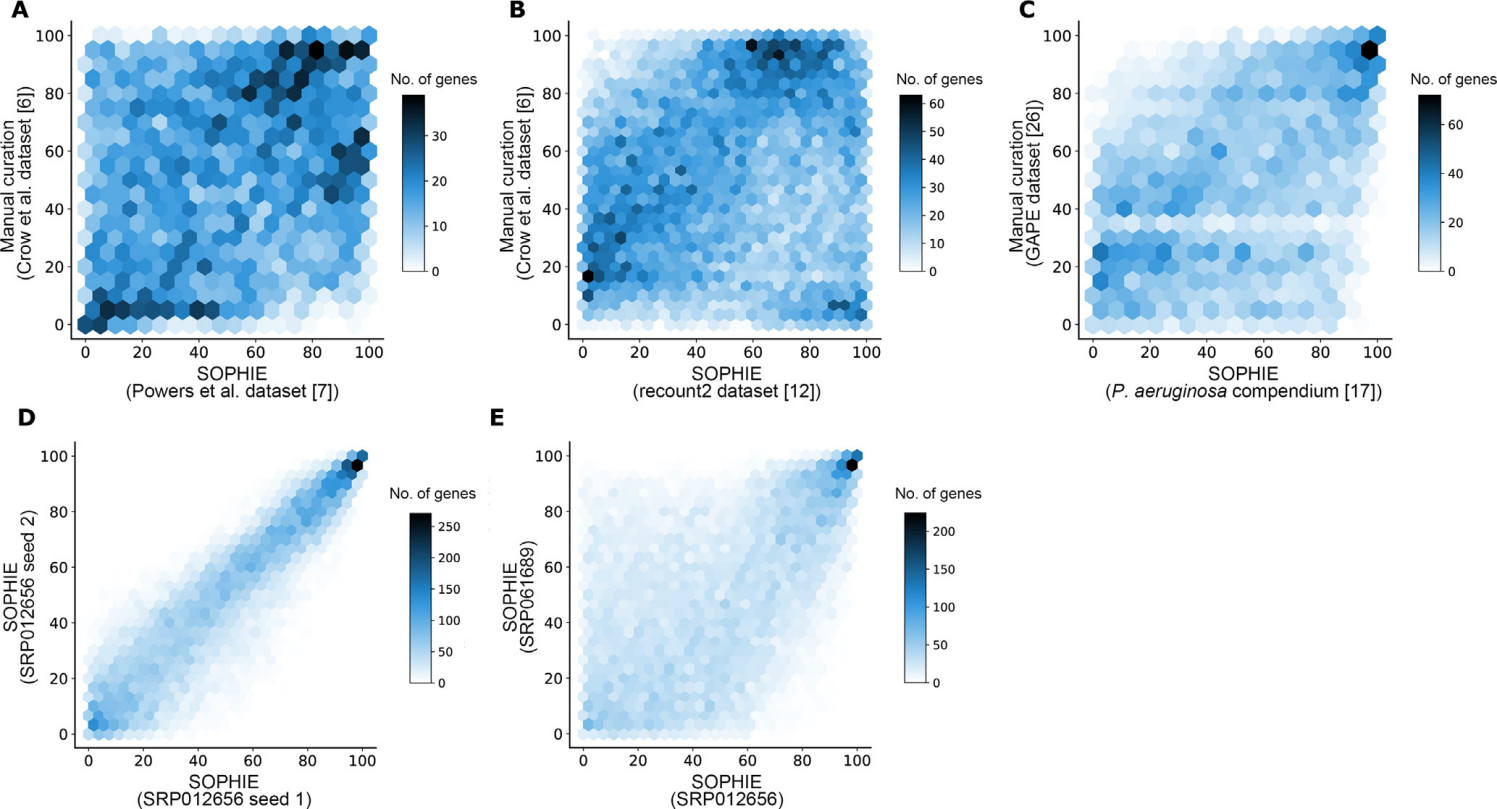
**Comparison of SOPHIE results with manual curation** **A.** Spearman correlation (R^2^ = 0.26) between gene percentiles obtained by our SOPHIE approach trained on the Powers et al. dataset (array) [7] using GSE11352 as a template (x-axis) and gene percentiles obtained by manually curated experiments from the Crow et al. dataset [6] (y-axis). The two datasets used the same array platform but different contexts. There is a significant (*P* = 2E−8) over-representation of SOPHIE-identified common DEGs within the common DEGs identified by Crow and colleagues [6]. **B.** Spearman correlation (R^2^ = 0.185) between gene percentiles obtained by our SOPHIE approach trained on the recount2 dataset (RNA-seq) [12] using SRP012656 as a template (x-axis) and gene percentiles obtained by manually curated experiments from the Crow et al. dataset (y-axis). There is a significant (*P* = 2E−15) over-representation of SOPHIE-identified common DEGs within the common DEGs identified by Crow and colleagues [6]. **C.** Spearman correlation (R^2^ = 0.45) between gene percentiles obtained by our SOPHIE approach trained on the *P. aeruginosa* compendium (array) [17] using E-GEOD-33245 as a template (x-axis) and gene percentiles obtained by manually curated experiments from GAPE [26] (y-axis). There is a significant (*P* = 1E−139) over-representation of SOPHIE-identified common DEGs within the common DEGs in the GAPE dataset. **D.** Spearman correlation (R^2^ = 0.907) between gene percentiles generated by SOPHIE using two runs of the same experiment (SRP012656). **E.** Spearman correlation (R^2^ = 0.572) between gene percentiles generated by SOPHIE using two different template experiments (SRP012656 and SRP061689). GAPE, GAUGE-annotated *Pseudomonas aeruginosa* and *Escherichia coli* transcriptomic compendia for reanalysis.

We next examined whether or not common DEGs were consistent across training datasets and platforms. We applied SOPHIE to a different collection of microarray data that accompanied another prior report of common differentially expressed pathways [7], which included 442 DE analyses (from 2812 human microarray datasets) testing the response of small-molecule treatments in cancer cell lines. For this analysis, we selected an arbitrary template experiment (GSE11352, which examined estradiol exposure in breast cancer cells [35]) to generate simulated experiments. We calculated DE statistics for each experiment and then calculated the percentile of genes by their median log_2_ FC across the simulated experiments. We found concordance between the percentiles from SOPHIE-identified common DEGs using a VAE trained on the Powers et al. dataset and those from the Crow et al. results using Spearman correlation (Figure 2A). The concordance was particularly high for the genes in the highest and lowest percentiles, *i.e.*, the most and least common DEGs, respectively. Furthermore, there was a significant (*P* = 2E−8) over-representation of SOPHIE-identified common DEGs within the common DEGs identified by Crow and colleagues [6]. While the two datasets used the same array platform to generate data, the datasets have different compositions — the Crow et al. dataset is a heterogenous mixture of different types of experiments while the Power et al. dataset is cancer cell lines specifically treated with small molecules. Despite the differences in context, the consistency observed in the common DEGs demonstrates that many common DEGs are differentially expressed regardless of the context.

In general, transcriptome analysis approaches can be difficult to translate between different platforms (RNA-seq and microarray) and datasets. To demonstrate whether common DEGs were consistent across platforms, we applied SOPHIE to human RNA-seq data from recount2 [12]. We selected an arbitrary template experiment from recount2 (SRP012656, which examined non-small cell lung adenocarcinoma tumors [36]), simulated experiments, and calculated DEGs using DESeq2. For this template experiment, primary non-small cell lung adenocarcinoma tumors were compared to adjacent normal tissues for 6 never-smoker Korean female patients. We again examined concordance between the gene percentiles from the SOPHIE results and those from the Crow et al. results (Figure 2B). Althought the Crow et al. dataset was measured on microarrays and recount2 used an RNA-seq platform, we still found a significant (*P* = 2E−15) over-representation of SOPHIE-identified common DEGs shared with the Crow et al. results.

We also noticed a set of genes in the bottom right corner of Figure 2B with a high percentile score that were common DEGs in RNA-seq data but not in the Crow et al. data. We did not observe a corresponding set in the upper left corner, suggesting that RNA-seq captures the microarray-based common DEGs, but prior microarray-based reports lack certain RNA-seq-specific ones. This subset of genes was identified to be commonly differentially expressed in RNA-seq data and not in array data, suggesting that platform differences underlie this effect. Some preliminary experiments showed that common DEGs identified specifically in the RNA-seq data tended to have a lower expression compared to those identified using both the array and RNA-seq platforms (Figure S2). The VAE, used by ponyo in the simulation step, appeared to artificially boost the expression of these RNA-seq-identified common DEGs, so that they were found to be differentially expressed. Unlike the array data, the RNA-seq data had a larger variance and so the effects of the VAE were more pronounced, affecting genes in the outliers of the compendium distribution, which included these RNA-seq-identified common DEGs. In general, a consistent set of common DEGs was found using two datasets that had similar contexts — they both contained a mixture of different types of experiments — but used different platforms. This consistency indicates that there are some common DEGs that were differentially expressed across different platforms.

Overall, using SOPHIE we find that there exist some common DEGs that are consistent across contexts and platforms, *i.e.*, there is a set of common DEGs, regardless of context or platform (Figure S3).

### SOPHIE generalized to other organisms

Finally, when we extended SOPHIE to a different organism, *P. aeruginosa*, we observed concordance (R^2^ = 0.449) between SOPHIE-generated percentiles and those generated using a manually curated dataset, GAPE [26] (Figure 2C). GAPE contained a collection of 73 array experiments from the GPL84 platform. GAPE performed automatic group assignments of those experiments that were then manually verified by human curators. We then calculated the percentile for how frequently genes were differentially expressed across the 73 experiments. For this analysis, we selected the template experiment E-GEOD-33245, which examined different targets of the carbon catabolite control system [14], to generate simulated experiments. We calculated DE statistics for each experiment and then calculated the percentile of genes by their median log_2_ FC across the simulated experiments. We found a significant (*P* = 1E−139) over-representation of SOPHIE-identified common DEGs within the common DEGs in the GAPE dataset. Again, without any curation, SOPHIE recapitulated the common findings reported in the GAPE dataset, which was generated using a manually curated approach. Considering our previous results using human data, the consistency found in these results demonstrates the generalizability of SOPHIE to other organisms like bacteria, *i.e.*, using our SOPHIE approach we could easily switch out the human training dataset with a bacterial one.

### SOPHIE common findings were robust

Having shown that SOPHIE can recapitulate the percentiles of common DEGs identified by two manually curated datasets (Crow et al. or GAPE) using a variety of input datasets, we next examined the robustness of these common patterns using a human compendium. We compared SOPHIE-generated percentiles from different simulations using the same template experiment and found a very strong correlation (R^2^ = 0.907), especially for high- and low-percentile genes (Figure 2D). The genes in the middle percentiles were more sensitive to changes, so the signal was less clear; however, this was not unexpected with rank-based analysis in gene expression, where small changes near the middle of the distribution can produce large differences in rank. This noise was more pronounced when we compared the percentiles generated by SOPHIE using two different template experiments (Figure 2E). In addition, we observed that common DEGs were consistent across different template experiments (R^2^ = 0.572). Overall, SOPHIE common findings were robust to different runs and template experiments selected.

### Common differentially expressed pathways identified by SOPHIE recapitulated the curation-derived ones

**Figure 3:**
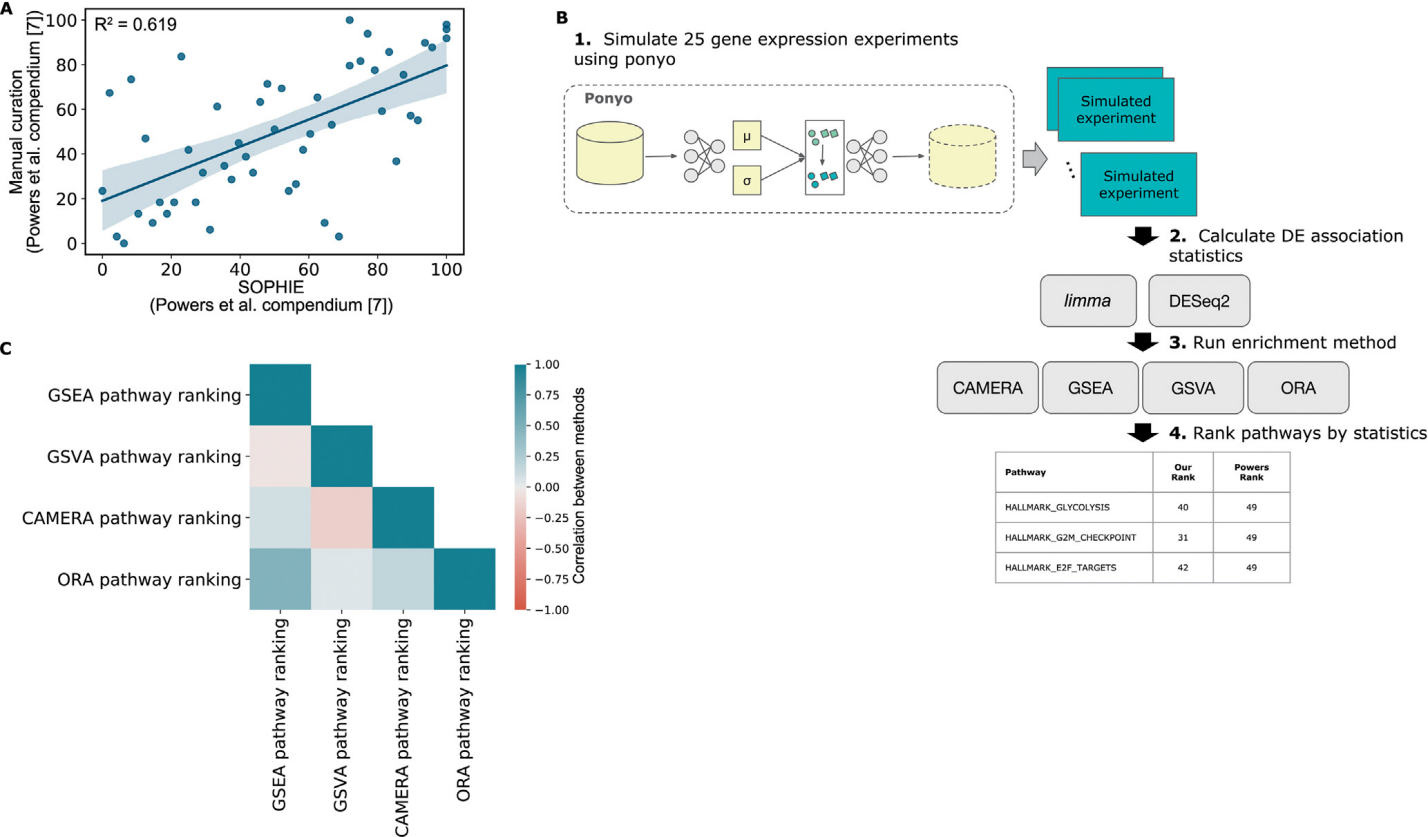
**Application of SOPHIE to pathways** **A.** Correlation between pathway percentiles determined by our simulated method trained on the Powers et al. compendium [7] (x-axis) and pathway percentiles obtained by a manual curation approach from the same Powers et al. compendium [7] (y-axis). **B.** Workflow describing how the SOPHIE pipeline can be easily extended to plug in different enrichment methods. **C.** Correlation of pathway percentiles between different enrichment methods (GSEA, GSVA, CAMERA, and ORA) using RNA-seq data. GSEA, gene set enrichment analysis; GSVA, gene set variation analysis; CAMERA, correlation adjusted mean rank gene set test; ORA, over-representation analysis.

In addition to common DEGs, we also examined common differentially expressed pathways. While there is some variation between the ranking of common DEGs, grouping genes into pathways may find more robust common signals. For this analysis we used a set of common differentially expressed pathways reported by Powers and colleagues [7]. We calculated the percentile per pathway by how frequently enriched they were across the 442 experiments. Then, similar to the previous analyses, we applied SOPHIE to the same Powers et al. data. We simulated 25 new experiments from the same template experiment used previously (GSE11352) and calculated DE statistics for each experiment. For this analysis, since we focused on pathways, we used GSEA [20] to identify pathways enriched in DEGs. We compared the percentiles of pathways determined using data simulated from SOPHIE with those we calculated based on the reported by Powers et al*.* [7] and found strong concordance (R^2^ = 0.619, Figure 3A). SOPHIE recapitulated the commonly enriched pathways reported by Powers et al. [7], which were obtained by a manual curation approach.

SOPHIE can also be applied using other pathway analysis methods. We easily extended SOPHIE to use multiple different enrichment methods (Figure 3B) and examined the common findings. We selected 4 enrichment methods (GSEA, GSVA, CAMERA, and ORA) from the study by Geistlinger and colleagues [37]. We selected methods if 1) that could be applied to both RNA-seq and array data and 2) that covered a wide range of statistical performance measures including runtime, the number of gene sets found to be statistically significant, and the type of method — self-contained *vs.* competitive. Overall, the percentile of common pathways enriched varied between enrichment methods, likely due to the different assumptions and modeling procedures (Figure 3C, Figure S4). Therefore, scientists will need to use a method-specific common correction approach. Similar to our analysis of common DEGs, compared to the manual curation approach by Powers et al. [7], SOPHIE can automatically identify commonly changed pathways. Additionally, SOPHIE can be easily customized to use different enrichment methods depending on the analysis.

### Common DEGs may correspond to hyperresponsive pathways

**Figure 4:**
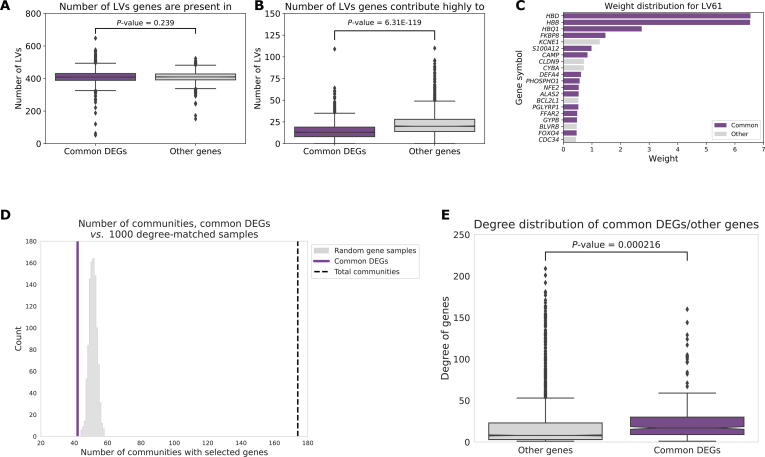
**Characterization of common DEGs** **A.** Number of human PLIER LVs in which common DEGs and other genes are present (*P* = 0.239, *t*-test). **B.** Number of human PLIER LVs in which common DEGs and other genes have a high weight score (*P* = 6.31E−119, *t*-test). **C.** Distribution of top-weighted human genes in example LV61, which was found to contain a high proportion of high-weight common DEGs. **D.** The number of communities with at least one commonly changed *P. aeruginosa* gene (purple) compared to the distribution of the number of communities with at least one non-commonly changed gene across 1000 samplings (gray) with the total number of communities marked by the black dashed line. **E.** Degree distribution of commonly changed *P. aeruginosa* genes (purple) compared to other genes (gray). PLIER, pathway-level information extractoR; LV, latent variable.

We next examined how the genes that were commonly differentially expressed were related to previously reported transcriptional patterns, the PLIER model [27], to gain insight into the role of these common DEGs. We identified common DEGs using recount2, which is a heterogeneous compendium of human gene expression data containing a range of different types of experiments and tissue types. The recount2 data were decomposed into PLIER LVs, representing gene expression modules, some of which were aligned with known curated pathways, in prior work [27]. In these LVs, genes had some weighted contribution, and we found that the median number of genes with a non-zero weight was 2824. We divided genes into a set of common DEGs, which were genes that were at the 60th percentile and above in our recount2 analysis (Figure 2B), and all other genes. We found that the number of LVs in which common DEGs were present was roughly the same as the number of LVs in which other genes (*i.e.*, non-common DEGs) were present (Figure 4A; *P* = 0.239, comparing the median numbers between the two gene groups). However, common DEGs were found among the highest weights (the 98th percentile and above for each LV) for fewer LVs than other genes (Figure 4B; *P* = 6.31E−119, comparing the median numbers of highly contributing genes between common DEGs and other genes). Taken together, these results suggested that common DEGs contributed to as many LVs as other genes (*i.e*., having a non-zero weight), but common DEGs occurred less frequently among the genes with highest weights. Overall, the wide coverage across PLIER LVs but lack of high weight contributions suggested that common DEGs across human experiments mainly contributed to a few pathways.

Given the small number of LVs in which common DEGs had high weights, one possibility for why these genes were commonly changed might be related to membership in a few hyperresponsive pathways. Since these LVs tend to be associated with particular biological processes, we tested if there were any LVs, and thereby processes, that contained a large fraction of common DEGs. If there exist LVs that were primarily composed of common DEGs, this might lend insight into the role of common DEGs. For this analysis, we ranked LVs by the proportion of commonly shifted genes at the 98th percentile and above. Overall, many of these LVs were associated with immune responses, signaling, and metabolism. One example LV, which contained a high proportion of common DEGs compared to other genes (proportion of common DEGs > 0.5), was LV61 (Figure 4C; Table S1). This LV included pathways related to immune response (neutrophils), signaling (biosynthesis of erythromycin – ERY2), and wound healing (megakaryocyte platelet production).

We performed a similar analysis to examine common patterns in the *P. aeruginosa* compendium. Again, we leveraged an existing model. Tan et al. previously created a low-dimensional representation of the *P. aeruginosa* compendium using a denoising autoencoder, called eADAGE, where some of the LVs were found to be associated with Kyoto Encylopedia of Genes and Genomes (KEGG) pathways and other biological sources of variation [17], [38], [39]. Using this existing eADAGE model, we created a gene–gene similarity network where the correlation within the eADAGE representation was used to connect genes. After performing a community detection analysis, we discovered that common DEGs, which had high concordance between SOPHIE and GAPE, tended to cluster in fewer communities compared to other genes (Figure 4D; Table S2). Furthermore, common DEGs had a higher median degree in the eADAGE similarity network compared to other genes (*P* = 2.16E−4; Figure 4E). These observations were consistent with an analysis that found a set of virulence-related transcriptional regulators that target multiple pathways [40]. Together, these results suggested that, like the patterns we observed in the human dataset, there were relatively few communities that common DEGs contributed strongly to. These few communities containing common DEGs were highly connected to other communities, again suggesting that certain pathways may be particularly responsive to perturbations.

### SOPHIE-identified common DEGs were involved in, but not specific to, the carbon catabolite repression system in *P. aeruginosa*

**Figure 5:**
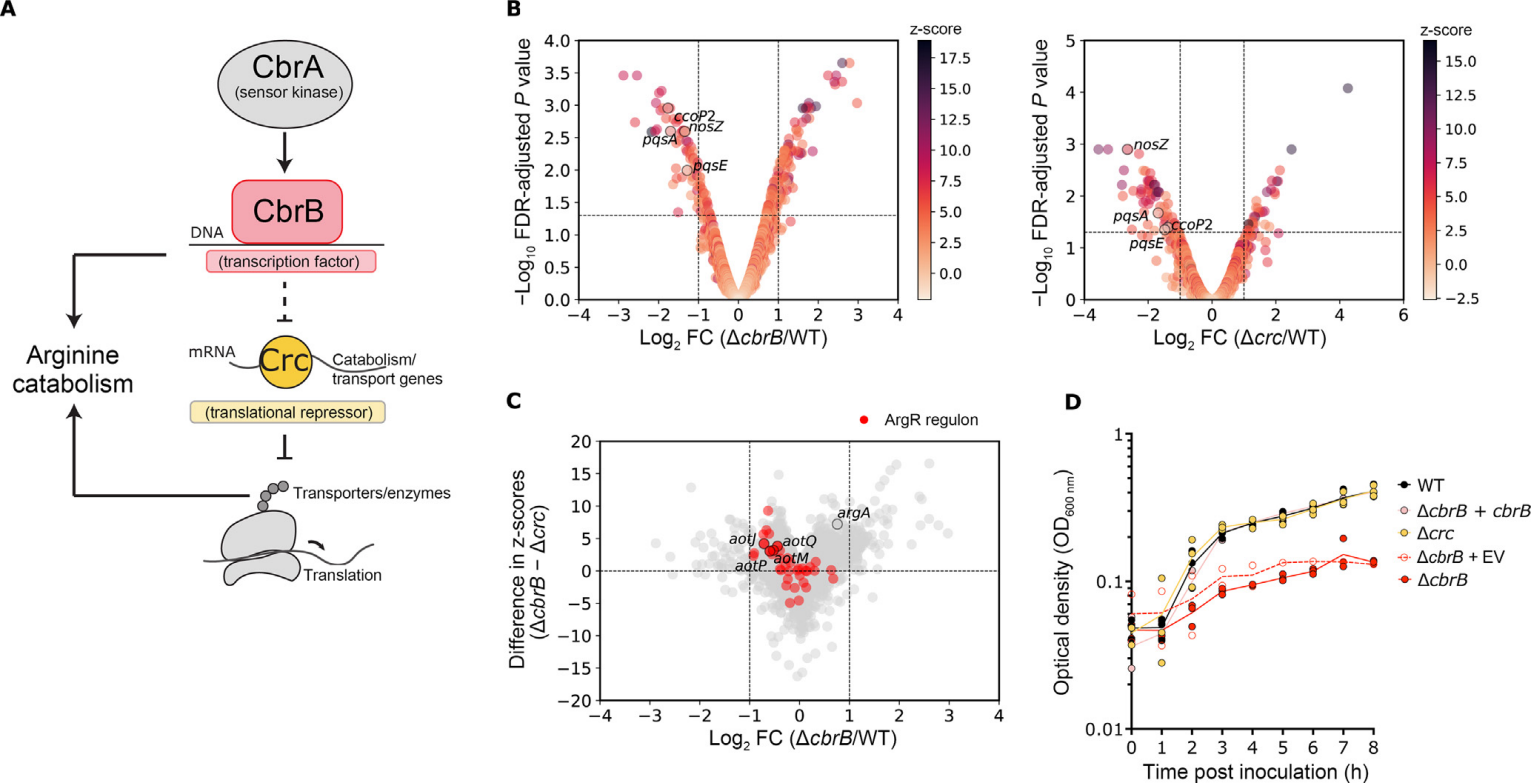
**Molecular validation of SOPHIE findings** SOPHIE can identify genes with specific expression shifts in experiments. **A.** Model of CbrAB system. **B.** Volcano plot with log_2_ FC *vs.* log_10_ FDR-adjusted *P* value for Δ*cbrB* mutant *vs.* WT and Δ*crc* mutant *vs.* WT. The darker hue indicates a higher z-score and therefore higher specificity for the context being tested. **C.** Plot with log_2_ FC in Δ*cbrB* mutant context on the x-axis and difference in z-score in Δ*cbrB* and Δ*crc* mutant contexts on the y-axis. Changes that are specific to Δ*cbrB* have positive y-values and changes specific to Δ*crc* have negative y-values. **D.** Growth curves for *P. aeruginosa* in 10 mM arginine using WT (filled black), Δ*cbrB* mutant (filled red), Δ*cbrB* mutant with an empty expression vector (Δ*cbrB* + EV; empty red), Δ*cbrB* mutant with extrachromosomal complementation (Δ*cbrB* + *cbrB*; filled pink), and Δ*crc* mutant (filled yellow). *cbrB* and *crc* were removed when plotting panels (B) and (C). WT, wild type; FDR, false discovery rate.

In general, DE analyses often aim to understand the genetic causes and downstream consequences of gene expression. However, using traditional *P* values and log_2_ FC criteria, such datasets often contain hundreds of genes, many of which are secondary to changes in the phenotype of interest. Using SOPHIE, we distinguish between common DEGs and those that are specific to the context of the experiment. As a test case, we examined the common and specific DEGs generated using the template experiment E-GEOD-33245 which investigated the metabolic decision-making process known as carbon catabolite repression, which is important for *P. aeruginosa* pathogenicity [41] (Figure 5A).

To separate common and context-specific DEGs, we used the z-score that compared the log_2_ FC of a gene in a template experiment to the mean log_2_ FC of the same gene across the background set of experiments. A low z-score indicated that there was no significant difference in how changed the gene was between the template and the background set and therefore these genes were predicted to be common DEGs.

Genes that had a low z-score, indicating a high likelihood of it being part of common response, were differentially expressed in many experiments across the *P. aeruginosa* datasets: genes considered commonly differentially expressed by SOPHIE and GAPE accounted for a substantial fraction of DEGs in Δ*cbrB*/WT and Δ*crc*/WT comparisons, respectively (Figure 5B). Both comparisons included the well-studied genes *pqsA*, *pqsE*, *nosZ*, and *ccoP2* as commonly differentially expressed. One DEG in the Δ*crc*/WT comparison was *arcB*, encoding an ornithine carbamoyltransferase involved in the arginine deiminase pathway that produces ornithine from arginine under low oxygen conditions*.* Based on SOPHIE analysis, this gene had a z-score of 1.09, suggesting that it is a common DEG. This assignment as a common DEG aligned well with the published GAPE analysis that found *arcB* to be differentially expressed in 40 out of the 73 annotated *P. aeruginosa* studies.

### SOPHIE identified arginine catabolism genes as specific to components in the carbon catabolite repression system

In addition to the identification of common DEGs, an orthogonal use of SOPHIE can be applied when analyzing experimental conditions that uncover unrecognized but specific genes of interest. In the analysis for separating common and specific DEGs, SOPHIE can highlight genes that show modest, but specific changes that would be missed by traditional DE analysis. This use is applicable to the carbon catabolite repression dataset (E-GEOD-33245) which includes investigations into multiple genetic components of the same molecular pathway that collectively controls metabolic decision-making. Ultimately, this pathway determines the order of metabolite consumption. This decision process depends on a complex molecular mechanism involving both transcriptional and translational regulations that results in both direct and indirect effects on the transcriptome, respectively. A previous analysis by Sonnleitner et al. [14] has suggested that the production of catabolic enzymes and transporters is controlled by the translational co-repressor Crc (Figure 5A). In the presence of non-repressive carbon sources, the CbrA kinase promotes the activity of the transcription factor CbrB, which directly modulates the levels of the small RNA *crcZ* among other transcripts*.* In turn, *crcZ* sequesters the Crc protein [42] thereby enabling translation to occur.

We focused on the comparisons between WT and isogenic Δ*cbrB* and Δ*crc* mutants from E-GEOD-33245 and sought to identify transcriptional changes specific to one or the other regulator. In the absence of the transcription factor CbrB or the translational co-repressor Crc, 156 and 149 genes were differentially expressed (|log_2_ FC| > 1, FDR-adjusted *P* < 0.05), respectively, relative to WT. To select context-specific DEGs, we again used the z-score that compared the log_2_ FC of a gene in a template experiment to the mean log_2_ FC of the same gene across the background set of experiments, this time selecting for large z-scores. If a z-score was large, then the gene was more differentially expressed in the template experiment compared to the background set of experiments and therefore predicted to be specific to the template experiment. In our case, we selected genes that had a large z-score and that were specific in one condition *vs.* the other, so our z-scores were not necessarily the largest overall. Depending on the use case, scientists will need to determine which z-scores are large enough given the contextual constraints to consider.

SOPHIE revealed genes involved in aerobic arginine metabolism (*i.e.*, *argA*) and arginine transport (*i.e.*, *aotJ*, *aotQ*, *aotM*, and *aotP*) changed by less than 2-fold in both samples. However, although CbrB and Crc are part of the same metabolic regulatory pathway, the specificity (highly ranked z-score; Table S3) was high in Δ*cbrB* mutant but not in Δ*crc* mutant. Broadly, genes regulated by the arginine responsive regulator ArgR were more specific to the deletion of *cbrB* than *crc* (Figure 5C; Table S3) [43]. We constructed two *P. aeruginosa* PA14 mutant strains, Δ*cbrB* and Δ*crc*. We found that only Δ*cbrB* was defective for growth on arginine likely the result of defective transport or catabolism (Figure 5D). This result supports the model that arginine metabolism is specifically regulated by CbrB, consistent with published data by other studies [44], [45], and highlights the utility of SOPHIE to drive the prioritization of genes for follow-up analysis of candidate DEGs. This method is particularly powerful for those genes that do not change very much but do so more than in the background simulated experiments (*i.e*., specific genes). It is appreciated that small expression changes can have biological significance, but we often choose not to pursue these genes because it is more difficult to study and follow low expression changes. However, SOPHIE provides strong confidence scores that highlight biologically important, but less studied genes for further analysis. By leveraging publicly available data, SOPHIE identified candidate specific genes. Independently, we experimentally validated that these genes played a specific role in the context of the template experiment. SOPHIE can therefore successfully predict biologically relevant gene targets that further our mechanistic understanding and drive future analyses.

## Discussion

We introduce an approach, SOPHIE, named after one of the main characters from Hayao Miyazaki’s animated film *Howl’s moving castle*. Sophie’s outward appearance as an old woman, despite being a young woman that has been cursed, demonstrates that initial observations can be misleading. This is the idea behind out approach, which allows users to identify specific gene expression signatures that can be masked by common background patterns.

SOPHIE automatically identified common DEGs and differentially expressed pathways using public gene expression compendia. SOPHIE returned consistent genes and pathways, by percentile, compared to previous results using both human [6], [7], [12] and bacterial [17] datasets. SOPHIE also found that many common DEGs were consistent across contexts and platforms. Furthermore, experimental validation confirmed a group of genes that SOPHIE predicted to show context-specific DE. In contrast to using a manually curated dataset, SOPHIE can be easily extended to generate a background distribution of experiments for any organism with public data available. These background experiments define a set of genes and pathways that are commonly changed across many different experimental conditions. These background sets of changes, provide context to individual experiments, highlighting specific gene expression changes and thus giving insight into mechanisms relevant to specific contexts including disease conditions.

Compared to prior work using manually curated datasets, which requires laborious manual grouping [6], [7], [26], SOPHIE demonstrates consistent results but uses an automated process. Even if curated DE experiments were available, scientists would need to perform the DE analysis for each experiment to generate a background distribution of gene rankings. In addition to reducing time spent on curation, SOPHIE provides the benefit of being able to support arbitrary experimental designs. Since SOPHIE generates a background compendium based on a selected template experiment, the background dataset corrects for having different experimental designs and magnitudes of change so that more subtle biological patterns can be discovered. Also, unlike the curated approach, SOPHIE considers multiple experiments in aggregate through the VAE training. Since the VAE captures shared relationships between genes across different conditions, then the experiments that are simulated based on this VAE representation can allow for the detection of specific changes within a set of genes belonging to the same pathway as we demonstrated in this study. In short, SOPHIE identifies the same common patterns but in a fast and scalable way. SOPHIE also identifies specific yet subtle patterns compared to using manual curation. However, there was a subset of genes that were specifically differentially expressed using SOPHIE but not found using the manually curated background. In one case, SOPHIE is using RNA-seq while the manually curated data are based on hybridization technology (microarray). Some initial experiments showed that this inconsistency is likely due to platform differences and how the VAE handled these two different data types. Overall, SOPHIE results are consistent with previous findings regardless of platform, but we also identified differences that might indicate there exists a hierarchy of common changes depending on the platform.

Building on the discovery of these common signals, we also examined the potential role of these common DEGs. These common DEGs appear to contribute to a small number of hyperresponsive pathways (Figure 4). This supports the observation that genes found to be differentially expressed across different contexts may not be informative about the experimental manipulation of interest. Therefore, considering specificity can be complementary to using log_2_ FC activity to study biological processes.

SOPHIE is a general approach relying on generative neural networks. Depending on the data type, there likely exists some optimal neural network architecture that preserves the underlying structure in the data. In our case, we examined SOPHIE with a VAE. VAEs can inappropriately reduce the variance in the data due to the normality assumption [46], potentially affecting the number of DEGs. However, while this limitation is known, Lee et al. [8] demonstrated that VAEs can still produce realistic experiments in this context. Based on this limitation, we used SOPHIE with percentile ranks, aligning with prior work from Crow et al. [6], instead of raw values to identify common DEGs. While using a VAE was successful at allowing us to identify common DEGs in our SOPHIE framework, other generative neural networks may be superior, and future work is needed to optimize and assess different types of generative neural networks to determine what model is most appropriate for a given dataset, data type, or measurement platform. In addition to varying the type of generative neural network used, there are also other possible functions we could use to generate data — instead of a linear shift in the latent space we could apply a rotation in the latent space. Overall, SOPHIE is a general approach where different steps of the workflow can be replaced, and further research is required to determine the effect of these replacements.

One limitation is that our template experiments are comprised of two conditions, but there are many different types of experiments (*e.g.*, time course). To determine if common DEGs vary based on experiment design, we would need to curate more experiments testing different experimental designs and determine how to group samples to perform a DE analysis or develop a new metric to define how many genes change. Another limitation to our study is that ponyo uses a random linear shift to simulate experiments. While this linear shift uses a location drawn from the known distribution of gene expression data, this shift currently doesn’t allow us to vary or shift along certain axes, such as tissue type or drug, which would require a deeper understanding of the latent space structure and what it captures. If ponyo could be extended to simulate background experiments along a specific axis, like tissue type or drug, we could ask if there are different sets of common DEGs that come up as we vary along specific axes. These questions can help lead to an improved understanding of common signals and the type of correction that might be needed.

SOPHIE is a powerful approach that can be used to drive how we study mechanisms underlying different cellular states and diseases. With SOPHIE, we can identify common DEGs that might be useful for diagnostic [47] and detection [48] purposes. We can also identify specific signals that point to possible treatment options [49]. In general, studies trying to uncover these genetic mechanisms tend to focus on prominent biological signals — those genes that are strongly differentially expressed. However, with SOPHIE we can start to glean information about those genes that are subtle but specifically relevant to the biology in question. Overall, SOPHIE is a practice that can complement existing traditional analyses to separate specific *vs.* common DEGs and differentially expressed pathways. These context-specific genes and pathways include both subtle changes that are largely unexplored and prominent changes that might point to areas of treatment and biomarker development. In general, SOPHIE can easily be applied across a range of different datasets to help drive discovery and further understanding of mechanisms.

The best way to use SOPHIE in practice will depend on the scientific question and the ease with which leads can be validated. The software associated with this study is available at GitHub (https://github.com/greenelab/generic-expression-patterns) and users can apply SOPHIE to their own analysis via https://github.com/greenelab/sophie.

## Conclusion

In this study, we have introduced a portable approach to distinguish between common and specific transcriptional signals using a compendium to auto-generate a null set. We applied SOPHIE to several different datasets and found a set of genes that were generally commonly differentially expressed across different biological contexts and technology platforms. We also validated that SOPHIE could predict subtle and specific transcriptional patterns. With the large number of significant genes found from traditional DE analyses, we have demonstrated that SOPHIE allows us to better interpret the results from those individual experiments and highlight changes that are specific to the tested context that can help further our understanding of the mechanism or point to new avenues of research. Since this approach uses simulated data, we only require a sufficient amount of un-curated gene expression data. Consequently, SOPHIE can be applied to different datasets or potentially other omes.

## Code availability

All scripts used in this study are available in the GitHub repository (https://github.com/greenelab/generic-expression-patterns) under an open-source license to facilitate the reproducibility of these findings (BSD 3-Clause). This repository was archived on zenodo (https://zenodo.org/record/6800152). The repository’s structure is described in the readme file. The notebooks that perform the validation experiment for common DEGs and differentially expressed pathways can be found in “human_general_array_analysis” (SOPHIE trained on Crow et al.), “human_general_analysis” (SOPHIE trained on recount2), “human_cancer_analysis” (SOPHIE trained on Powers et al.), and “pseudomonas_analysis” (SOPHIE trained on the *P. aeruginosa* compendium) directories. The notebooks that explore why genes are commonly differentially expressed can be found in “LV_analysis” directory. The notebooks for the network analysis can be found in the “network_analysis” directory. All supporting functions to run these notebooks can be found in the “generic_expression_patterns_modules” directory. The virtual environment was managed using conda (version 4.6.12), and the required libraries and packages are defined in the environment.yml file. Additionally, scripts to simulate gene expression experiments using the latent space shifting approach are available as a separate module, called ponyo, and can be installed from PyPi (https://github.com/greenelab/ponyo). The Readme file describes how users can re-run the analyses associated with this study. In order for users to utilize SOPHIE to analyze their own data, a separate repository including the necessary SOPHIE scripts and templates documenting how to run SOPHIE is available at https://github.com/greenelab/sophie. All simulations were run on a CPU.

## CRediT author statement

**Alexandra J. Lee:** Formal analysis, Investigation, Methodology, Project administration, Software, Validation, Visualization, Writing - original draft, Writing - review & editing. **Dallas L. Mould:** Formal analysis, Investigation, Visualization, Writing - original draft, Writing - review & editing. **Jake Crawford:** Formal analysis, Investigation, Software, Writing - review & editing. **Dongbo Hu:** Software, Writing - review & editing. **Rani K. Powers:** Resources, Writing - review & editing. **Georgia Doing:** Writing - review & editing. **James C. Costello:** Conceptualization, Funding acquisition, Supervision, Writing - review & editing. **Deborah A. Hogan:** Conceptualization, Funding acquisition, Supervision, Writing - review & editing. **Casey S. Greene:** Conceptualization, Funding acquisition, Supervision, Writing - original draft, Writing - review & editing. All authors have read and approved the final manuscript.

## Competing interests

The authors have declared no competing interests.

## Supplementary material

Supplementary Figure S1 **Evaluation of size of background compendium** Varying the number of simulated experiments used in the background set will yield similar results. The Spearman correlation between gene percentiles using our SOPHIE approach trained on Crow et al. (array) using GSE10281 as a template to generate 5 (**A**), 10 (**B**), 25 (**C**), 50 (**D**), and 100 (**E**) simulated experiments (x-axis) versus percentiles using manually curated experiments from the same Crow et al. (y-axis).

Supplementary Figure S2 **Exploration of common DEGs found only using RNA-seq** Common DEGs found in RNA-seq but not array data indicate platform-specific shifts. **A.** Average gene expression for all genes in Crow et al. array dataset (grey), genes commonly found to be changed in both RNA-seq using SOPHIE and array dataset using Crow et al. (dark blue), genes commonly found to be differentially expressed only in RNA-seq dataset (light blue). **B.** Average gene expression for all genes in recount2 RNA-seq dataset (grey), genes commonly found to be differentially expressed in both RNA-seq using SOPHIE and array dataset using Crow et al. (dark blue), genes commonly found to be differentially expressed only in RNA-seq dataset (light blue). **C.** Average gene expression of genes commonly found to be differentially expressed only in RNA-seq dataset in template experiment (grey) compared to simulated experiment (light blue). **D.** Average gene expression of genes commonly found to be shifted in both RNA-seq and array datasets in template experiment (grey) compared to simulated experiment (dark blue). DEGs, .

Supplementary Figure S3 **Consistency of common DEGs across context and compendia A.** Overlap of the top 20% most commonly changed genes identified using a heterogeneous compendium (Crow et al.) compared to using a cancer-specific compendium (Powers et al.). **B.** Overlap of the top 20% most commonly changed genes identified using a compendium composed of experiments measured on the array (Crow et al.) compared to one measured on RNA-seq (recount2).

Supplementary Figure S4 **Comparison of pathway ranking using different enrichment methods** Different pathway enrichment methods will find different commonly enriched pathways. Scatterplot showing the correlation of pathway percentiles between different enrichment methods (GSEA, GSVA, CAMERA, ORA) using RNA-seq data. GSEA, gene set enrichment analysis; GSVA, gene set variation analysis; CAMERA, correlation adjusted mean rank gene set test; ORA, over-representation analysis.
