# Supplementary material for: SOPHIE: Generative Neural Networks Separate Common and Specific Transcriptional Responses
Source: Genomics Proteomics Bioinformatics. 2022 Oct 7;20(5):912–27. doi: 10.1016/j.gpb.2022.09.011 (PMC10025681; doi:10.1016/j.gpb.2022.09.011)
Supplement: Supplementary Figure S1 — Evaluation of size of background compendium Varying the number of simulated experiments used in the background set will yield similar results. The Spearman correlation between gene percentiles using our SOPHIE approach trained on Crow et al. (array) using GSE10281 as a template to generate 5 (A), 10 (B), 25 (C), 50 (D), and 100 (E) simulated experiments (x-axis) versus percentiles using manually curated experiments from the same Crow et al. (y-axis). [file mmc1.pdf]

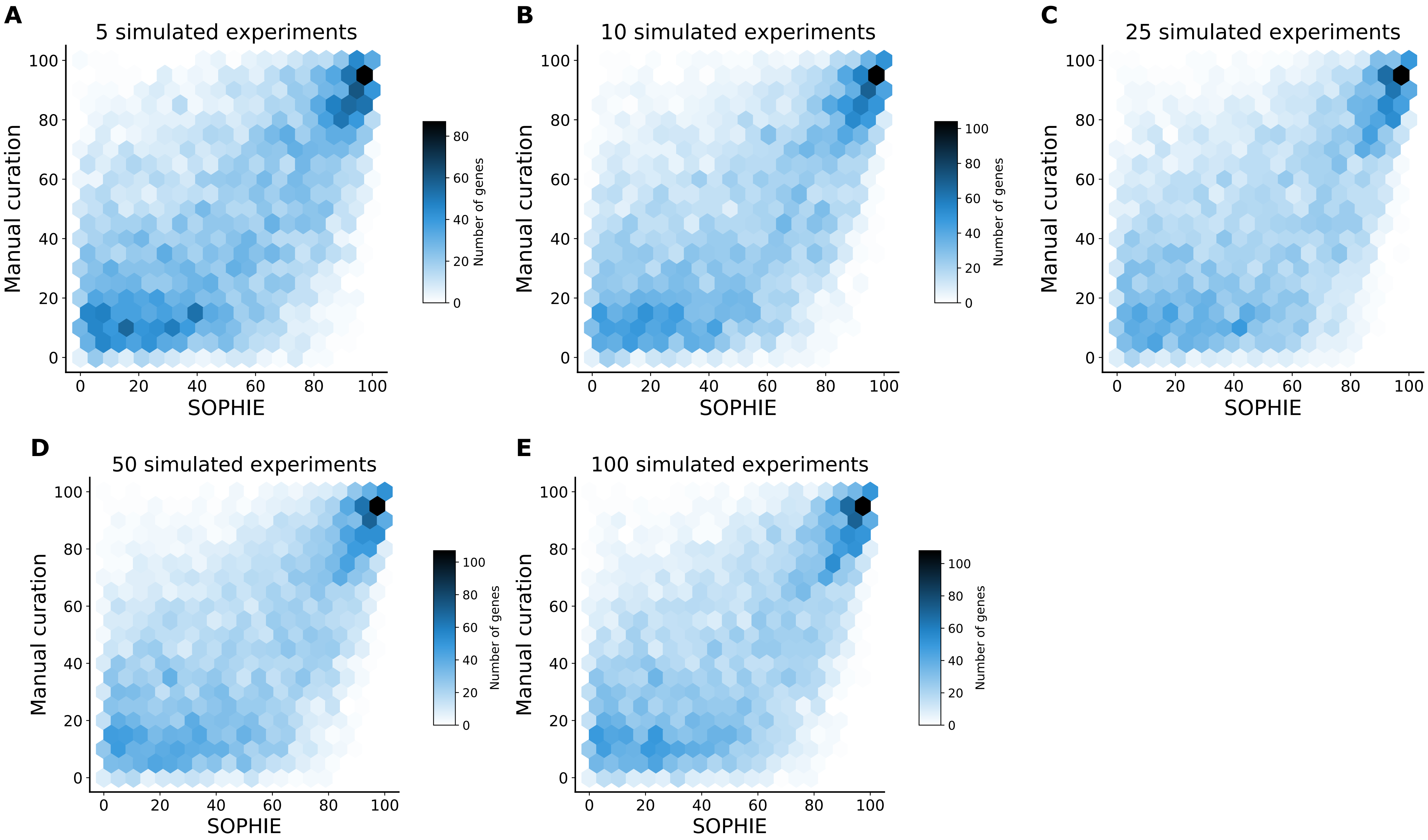

A
5 simulated experiments
Manual curation
SOPHIE
Number of genes
B
10 simulated experiments
Manual curation
SOPHIE
Number of genes
C
25 simulated experiments
Manual curation
SOPHIE
Number of genes
D
50 simulated experiments
Manual curation
SOPHIE
Number of genes
E
100 simulated experiments
Manual curation
SOPHIE
Number of genes
