# Supplementary figures and images for: SOPHIE: Generative Neural Networks Separate Common and Specific Transcriptional Responses
Source: Genomics Proteomics Bioinformatics. 2022 Oct 7;20(5):912–27. doi: 10.1016/j.gpb.2022.09.011 (PMC10025681; doi:10.1016/j.gpb.2022.09.011)

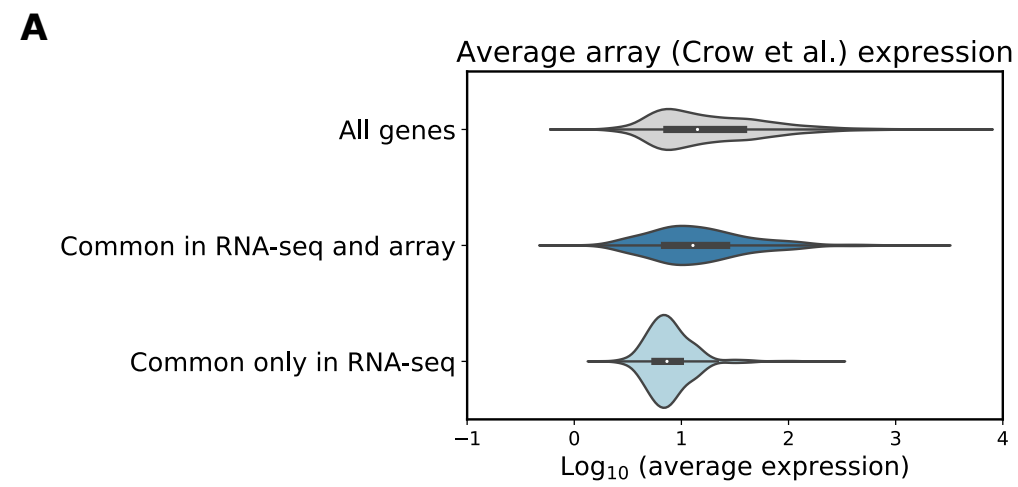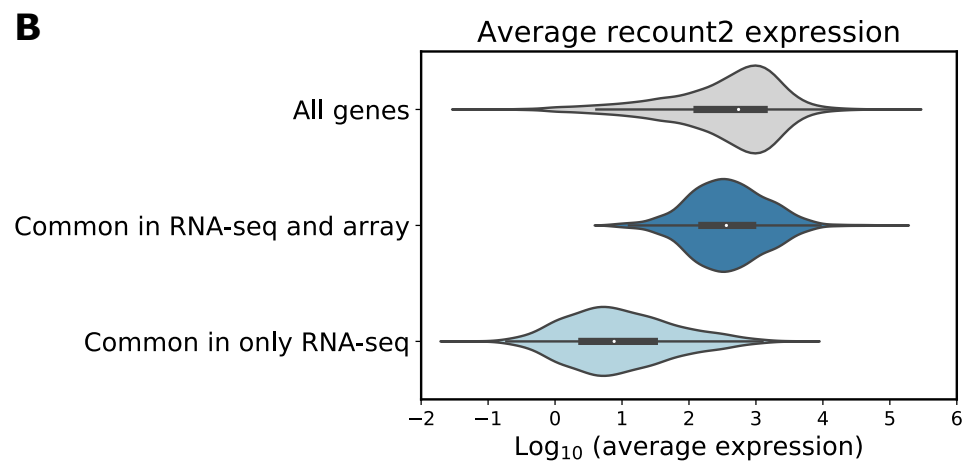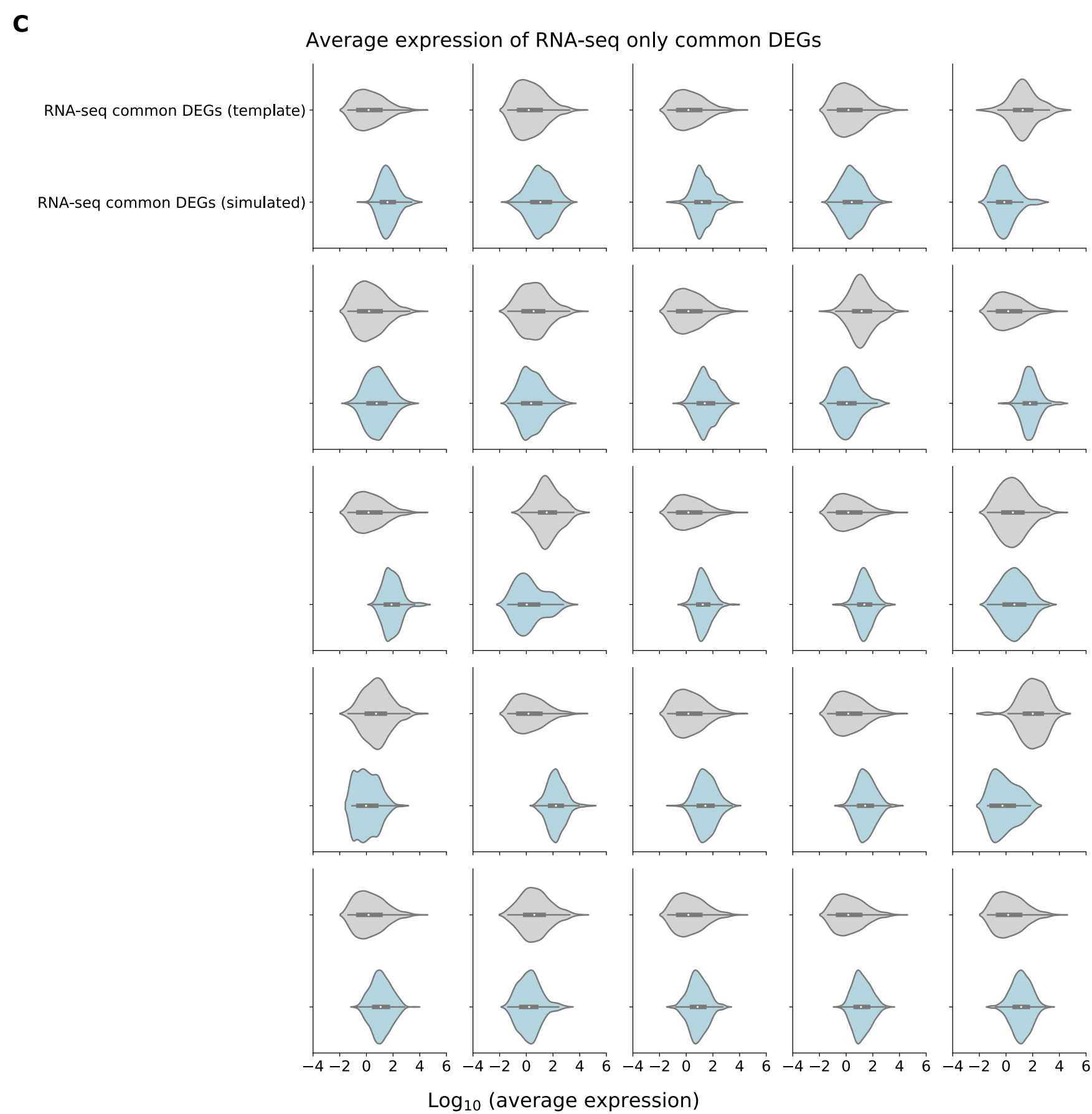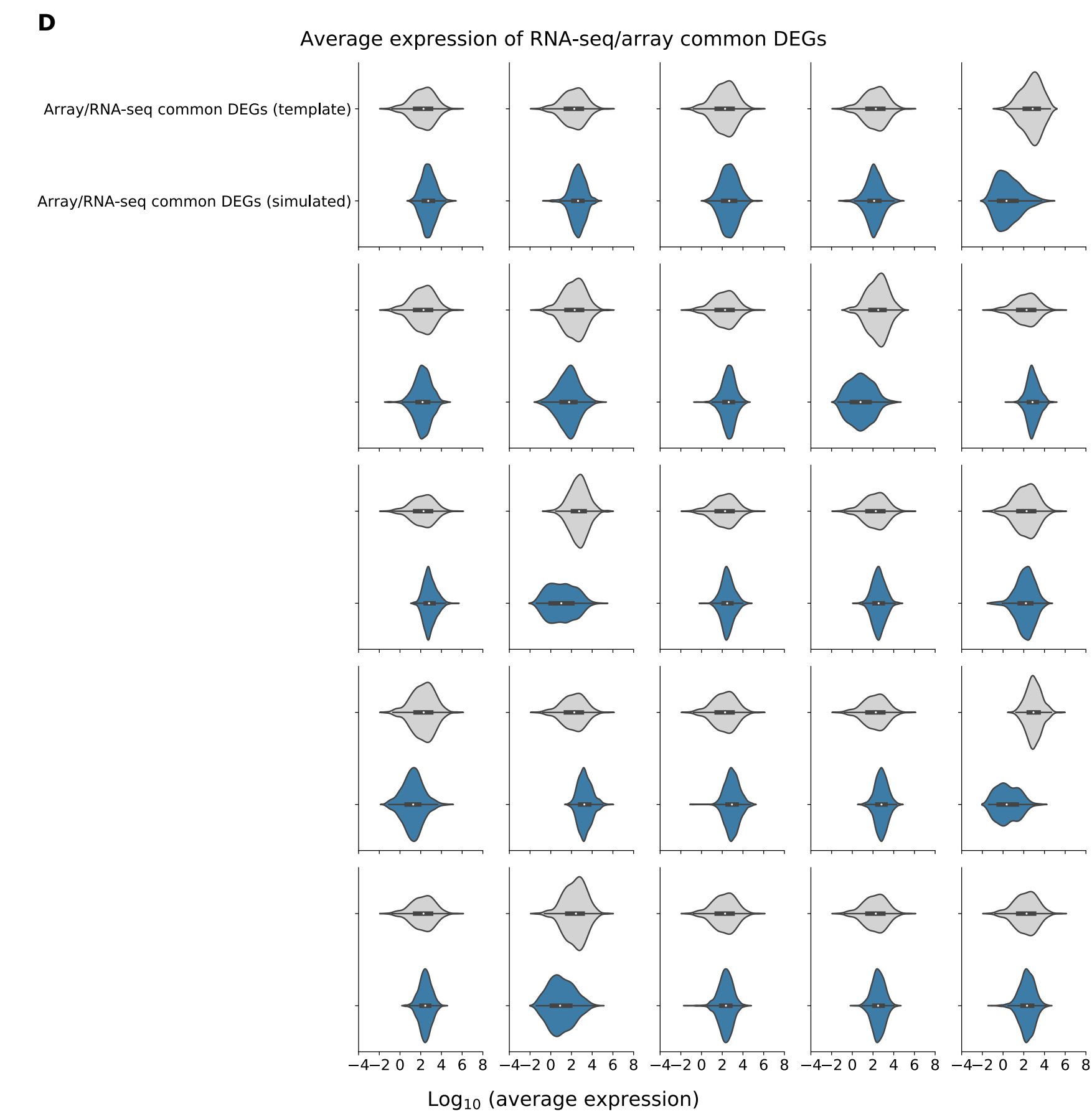

Supplement: Supplementary Figure S2 — Exploration of common DEGs found only using RNA-seq Common DEGs found in RNA-seq but not array data indicate platform-specific shifts. A. Average gene expression for all genes in Crow et al. array dataset (grey), genes commonly found to be changed in both RNA-seq using SOPHIE and array dataset using Crow et al. (dark blue), genes commonly found to be differentially expressed only in RNA-seq dataset (light blue). B. Average gene expression for all genes in recount2 RNA-seq dataset (grey), genes commonly found to be differentially expressed in both RNA-seq using SOPHIE and array dataset using Crow et al. (dark blue), genes commonly found to be differentially expressed only in RNA-seq dataset (light blue). C. Average gene expression of genes commonly found to be differentially expressed only in RNA-seq dataset in template experiment (grey) compared to simulated experiment (light blue). D. Average gene expression of genes commonly found to be shifted in both RNA-seq and array datasets in template experiment (grey) compared to simulated experiment (dark blue). DEGs, . [file mmc2.pdf]

**A**

Different context, same platforms

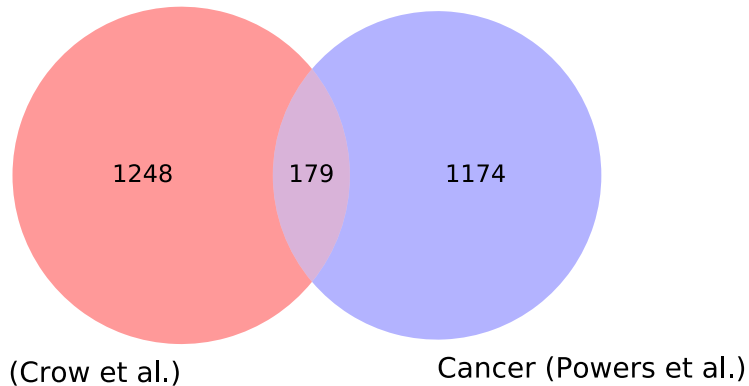**B**

Same context, different platforms

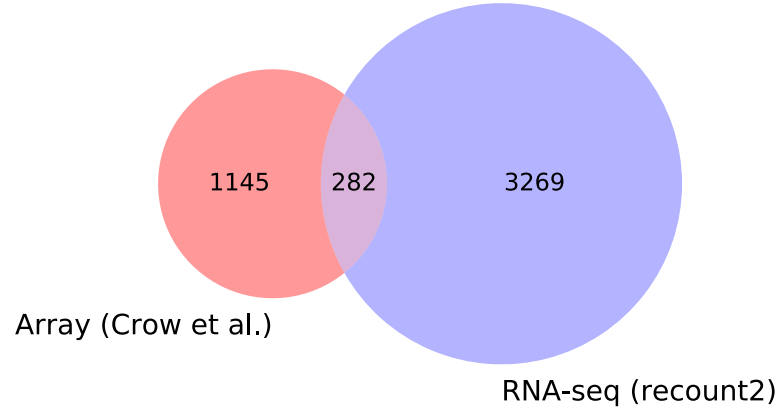

Supplement: Supplementary Figure S3 — Consistency of common DEGs across context and compendia A. Overlap of the top 20% most commonly changed genes identified using a heterogeneous compendium (Crow et al.) compared to using a cancer-specific compendium (Powers et al.). B. Overlap of the top 20% most commonly changed genes identified using a compendium composed of experiments measured on the array (Crow et al.) compared to one measured on RNA-seq (recount2). [file mmc3.pdf]

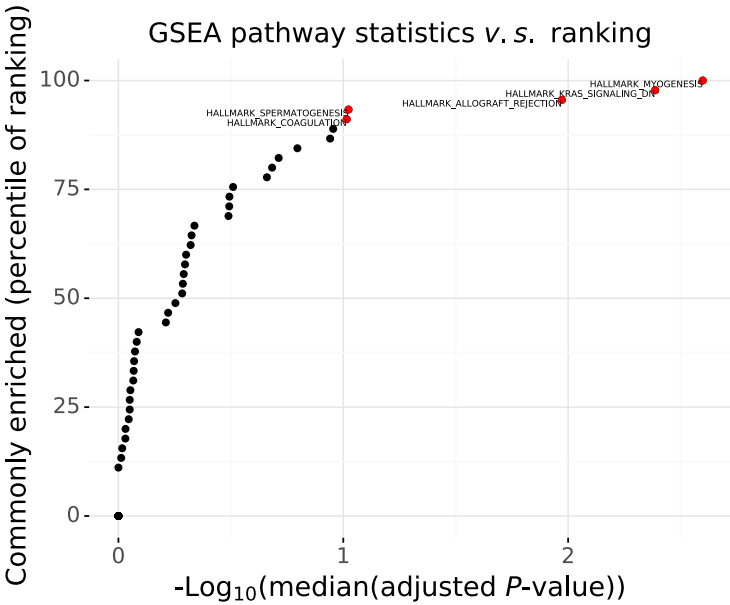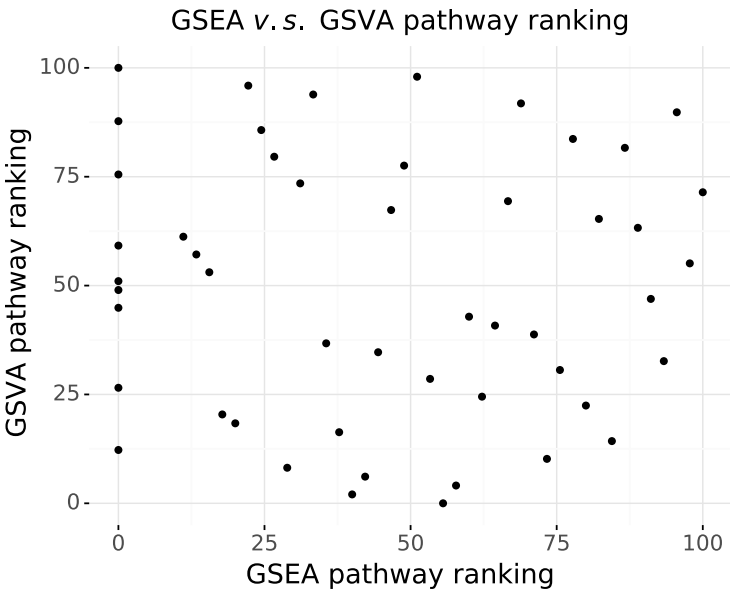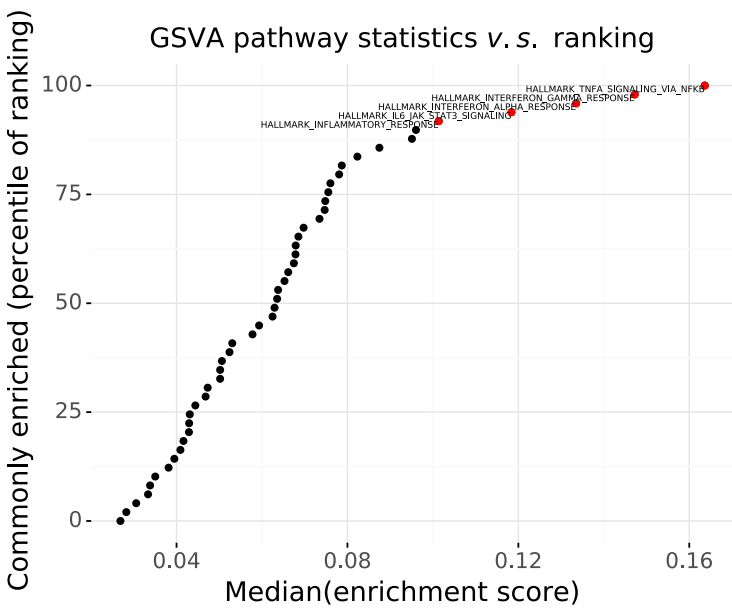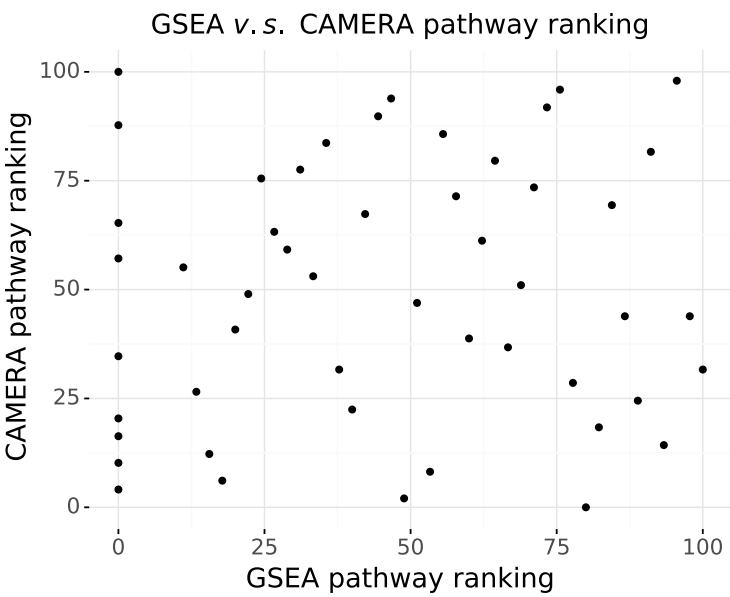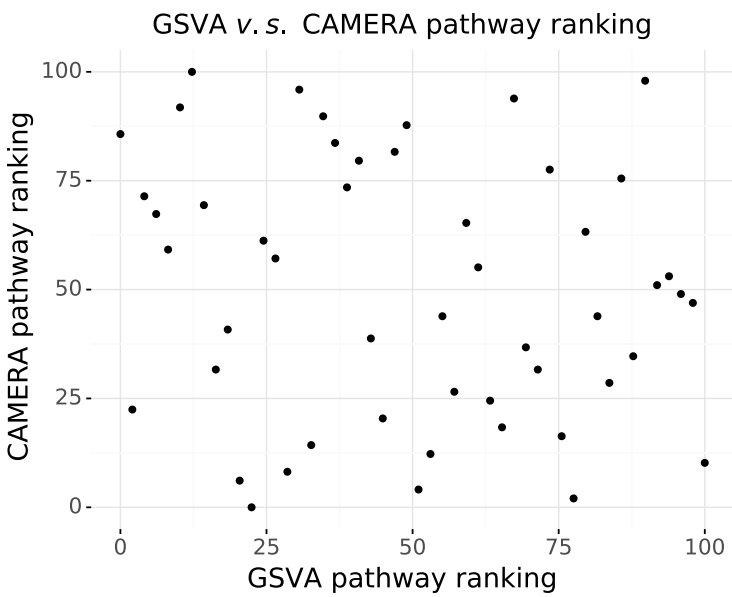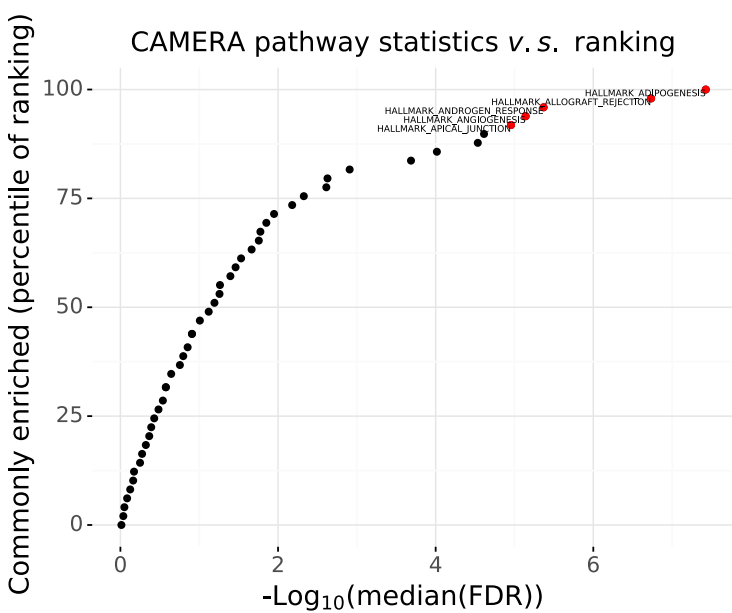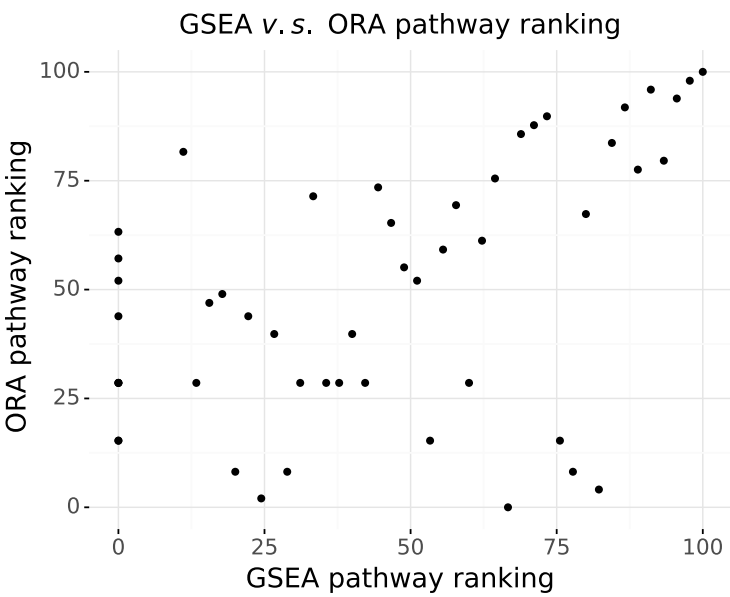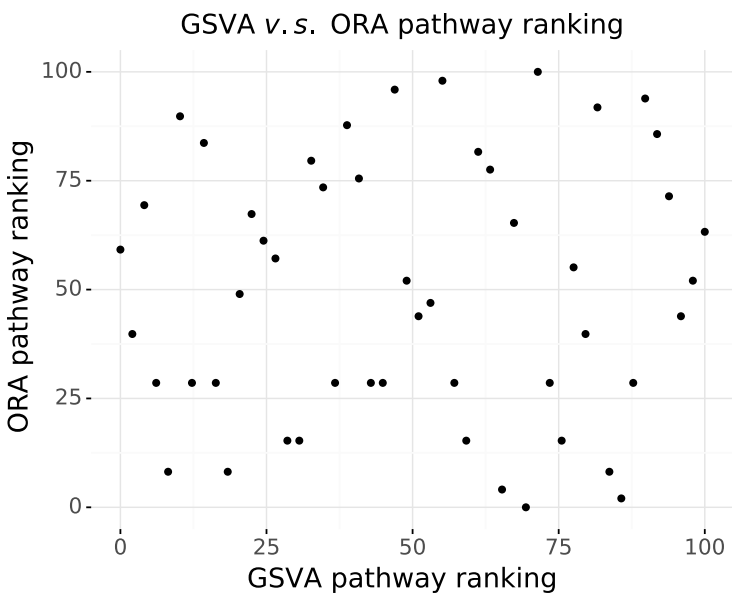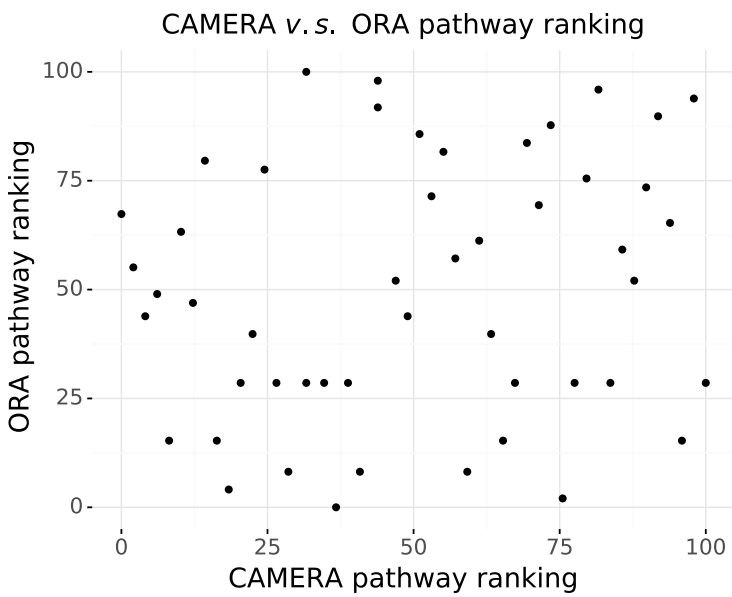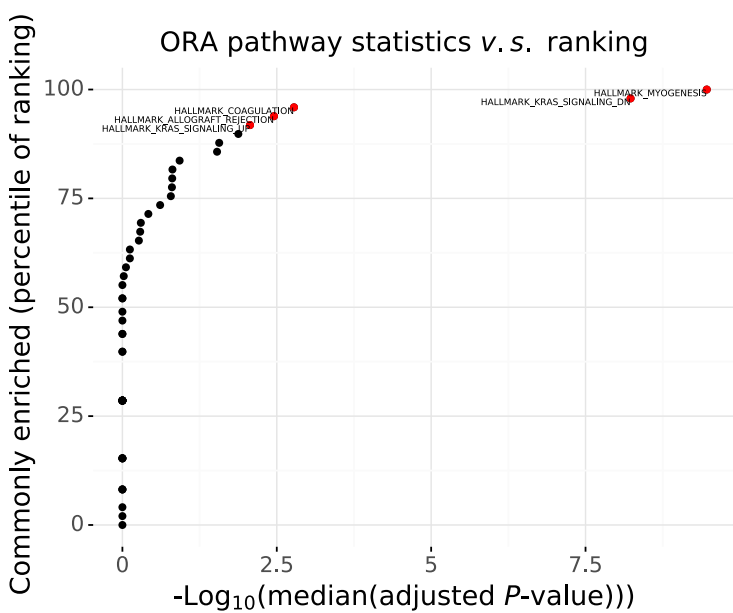

Supplement: Supplementary Figure S4 — Comparison of pathway ranking using different enrichment methods Different pathway enrichment methods will find different commonly enriched pathways. Scatterplot showing the correlation of pathway percentiles between different enrichment methods (GSEA, GSVA, CAMERA, ORA) using RNA-seq data. GSEA, gene set enrichment analysis; GSVA, gene set variation analysis; CAMERA, correlation adjusted mean rank gene set test; ORA, over-representation analysis. [file mmc4.pdf]
